# The Coordination of Leaf Photosynthesis Links C and N Fluxes in C_3_ Plant Species

**DOI:** 10.1371/journal.pone.0038345

**Published:** 2012-06-07

**Authors:** Vincent Maire, Pierre Martre, Jens Kattge, François Gastal, Gerd Esser, Sébastien Fontaine, Jean-François Soussana

**Affiliations:** 1 INRA, UR874 UREP, Clermont-Ferrand, France; 2 INRA, UMR1095 GDEC, Clermont-Ferrand, France; 3 Blaise Pascal University, UMR1095 GDEC, Aubière, France; 4 Max Planck Institute for Biogeochemistry, Jena, Germany; 5 INRA, UR4 P3F, Lusignan, France; 6 Justus Liebig University, Institute for Plant Ecology, Giessen, Germany; DOE Pacific Northwest National Laboratory, United States of America

## Abstract

Photosynthetic capacity is one of the most sensitive parameters in vegetation models and its relationship to leaf nitrogen content links the carbon and nitrogen cycles. Process understanding for reliably predicting photosynthetic capacity is still missing. To advance this understanding we have tested across C_3_ plant species the coordination hypothesis, which assumes nitrogen allocation to photosynthetic processes such that photosynthesis tends to be co-limited by ribulose-1,5-bisphosphate (RuBP) carboxylation and regeneration. The coordination hypothesis yields an analytical solution to predict photosynthetic capacity and calculate area-based leaf nitrogen content (*N*_a_). The resulting model linking leaf photosynthesis, stomata conductance and nitrogen investment provides testable hypotheses about the physiological regulation of these processes. Based on a dataset of 293 observations for 31 species grown under a range of environmental conditions, we confirm the coordination hypothesis: under mean environmental conditions experienced by leaves during the preceding month, RuBP carboxylation equals RuBP regeneration. We identify three key parameters for photosynthetic coordination: specific leaf area and two photosynthetic traits (*k_3_*, which modulates N investment and is the ratio of RuBP carboxylation/oxygenation capacity (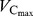) to leaf photosynthetic N content (*N*_pa_); and *J*_fac_, which modulates photosynthesis for a given *k*_3_ and is the ratio of RuBP regeneration capacity (*J*_max_) to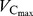). With species-specific parameter values of *SLA*, *k*_3_ and *J*_fac_, our leaf photosynthesis coordination model accounts for 93% of the total variance in *N_a_* across species and environmental conditions. A calibration by plant functional type of *k*_3_ and *J*_fac_ still leads to accurate model prediction of *N*_a_, while *SLA* calibration is essentially required at species level. Observed variations in *k_3_* and *J_fac_* are partly explained by environmental and phylogenetic constraints, while *SLA* variation is partly explained by phylogeny. These results open a new avenue for predicting photosynthetic capacity and leaf nitrogen content in vegetation models.

## Introduction

The response of leaf net photosynthesis to variations in light, temperature and CO_2_ concentration has been successfully represented by the biochemical model of C_3_ photosynthesis proposed by Farquhar, von Caemmerer and Berry [1]. This model has pioneered the mechanistic representation of the main biochemical processes of leaf photosynthesis, based on the assumption that photosynthesis is limited by either the carboxylation/oxygenation of ribulose-1,5-bisphosphate (RuBP) by the enzyme ribulose 1·5-bisphosphate carboxylase/oxygenase (Rubisco; *W*_c_), or the regeneration of RuBP by the electron transport chain (*W*_j_). Maximum rates of these two processes are determined by carboxylation capacity (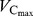) and electron transport capacity (*J*_max_). A strong correlation linearly links the variations of 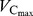 and *J*_max_ across species (*e.g.* [2]) and environmental conditions during plant growth (*e.g.* [3], [4]). Since both capacities are measured independently, this result suggests that CO_2_ assimilation is regulated in a coordinated manner by these two processes [5].

The variations of net photosynthesis with growth condition, season and species, are related to concurrent changes in leaf nitrogen content (*N*_a_) and to the allocation of nitrogen between different protein pools [6]. 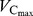 and *J*_max_ linearly correlate with *N*_a_ at both intra-and-interspecific levels [3], [4], [7]. Nevertheless, so far the relationship between 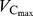 and *J*_max_ and their link to *N*_a_ are empirical correlations, their scatter is substantial, and a predictive process understanding C–N coupling at the leaf scale is still missing. As photosynthetic capacity is among the most influential parameters in current vegetation models [8], such an understanding is essential to predict photosynthesis at leaf, plant, stand and ecosystem scales under changing environmental conditions.

Haxeltine and Prentice [9] suggested a general model for the light-use efficiency of primary production, which links photosynthetic capacity and *N*_a_. This model is based on the Farquhar’s model of photosynthesis and has been implemented in the global terrestrial vegetation model LPJ [10]. This approach does not account for N limitation and is based on the optimization theory that maximizes assimilation against incoming radiation. Until now, a clear understanding of leaf N variations along vegetative canopies as well as across species and environments has not been provided by the optimization theory [11], [12]. For instance, all reported studies observed N gradients less steep than predicted with the optimization theory, suggesting that it likely overestimates predicted C gain [13]–[18]. Moreover, there are several limitations in optimization theory calculations (for a detailed discussion, see [19]).

Chen et al. [20] proposed an alternative approach: the coordination hypothesis of leaf photosynthesis. The basic assumption of this approach is that 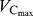 and *J*_max_ are actively regulated by plants in response to environmental conditions such that for most representative conditions *W*_c_ equals *W*_j_. The optimality criterion in this context is not maximum C gain (as proposed in [21]–[23]), but the balance of RuBP carboxylation and regeneration, providing a coordinated allocation of resources, *i.e.* nitrogen, to these two photosynthetic processes (Fig. S1). For vertical gradients within canopies the co-limiting N content was shown to increase with irradiance and to decline with temperature and with atmospheric CO_2_ concentration [20]. In agreement with experimental studies, the coordination hypothesis showed that N distribution with canopy depth declines less than the light gradient [13]–[18].

However, so far this co-limitation and its link to *N*_a_ has been considered only for vertical gradients within plant canopies, and has not yet been studied and validated across plant species and environmental conditions. This is possibly due to a lack of appropriate data including environmental growth conditions and photosynthetic parameters for a range of C_3_ plant species. In addition, a full test of this hypothesis requires extending the calculation of the co-limiting N content to account for the coupling between leaf photosynthesis and stomatal conductance [3] as well as ascribing leaf N to structural and metabolic pools [24], [25].

In this study, we evaluate for the first time the coordination hypothesis for sunlit leaves and its link to *N*_a_ for a large range of plant species grown under different environmental conditions. We use an extended version of the Farquhar model of C_3_ photosynthesis, a stomatal conductance model and a leaf N model to couple C, N and water fluxes at the leaf scale (see equations and variables in Tables 1–2). We apply this model to a dataset that includes leaf and environmental characteristics during plant growth and gas exchange measurements for a total of 31 C_3_ species (293 observations, Table S1). For each observation, plant characteristics included the specific leaf area (*SLA*, m^2^ ****g**^−^** ^1^ DM), *N*_a_ (gN****m**^−^** ^2^), and 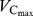 and *J*_max_ (µmol****m**^−^** ^2^ s**^−^** ^1^) at reference temperature and atmospheric CO_2_ concentration. The dataset covers six plant functional types (PFTs) grown both under constant and outdoors environments at a range of N and water supplies and atmospheric CO_2_ concentrations.

**Table 1:** Equations of the photosynthesis - stomatal conductance models.

Process	Equation	Unit	Eqn	Ref.
**Nitrogen sub-model**				
Leaf nitrogen content	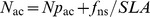	g N****m**^−^** ^2^	1	−
Leaf photosynthetic N content	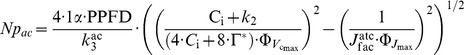	g N****m**^−^** ^2^	2	−
**Photosynthetic sub-model**				
Net photosynthetic rate	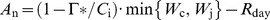	µmol m**^−^** ^2^ s**^−^** ^1^	3	[1]
Rubisco limited photosynthetic rate through RuBP carboxylation/oxygenation	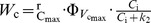	µmol m**^−^** ^2^ s**^−^** ^1^	4	[1]
Intermediate variable synthesising the Rubisco affinity for CO_2_	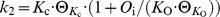	Pa	5	[1]
Maximum rate of carboxylation	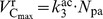	µmol m**^−^** ^2^ s**^−^** ^1^	6	[2]
RuBP regeneration limited photosyn**^−^**thetic rate through electron transport	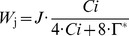	µmol m**^−^** ^2^ s**^−^** ^1^	7	[1]
Light dependence of electron transport rate	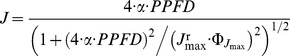	µmol m**^−^** ^2^ s**^−^** ^1^	8	[1]
Potential RuBP regeneration rate	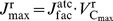	µmol m**^−^** ^2^ s**^−^** ^1^	9	[2]
CO_2_ compensation point in the absence of mitochondrial respiration	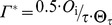	Pa	10	[1]
Leaf respiration without photorespiration	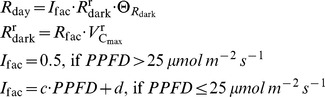	µmol m**^−^** ^2^ s**^−^** ^1^	11	[27]
Temperature dependence of *J*_max_ and *V*c_max_	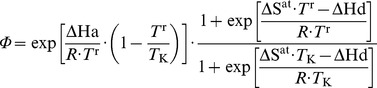	dimensionless	12	[31]
Temperature dependence of *K*_c_, *K*_o_, τ and *R*_dark_	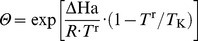	dimensionless	13	[27]
**Stomatal conductance sub-model**				
Stomatal conductance	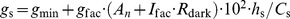	mmol m**^−^** ^2^ s**^−^** ^1^	14	[27]
CO_2_ partial pressure at the leaf boundary layer	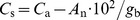	Pa	15	[3]
**Photosynthesis-stomata coupling**				
CO_2_ intercellular concentration	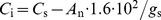	Pa	16	[29]
Analytical solution for photosynthesis calculation	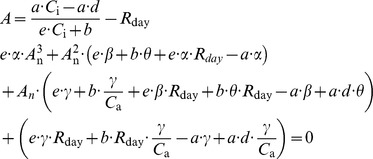 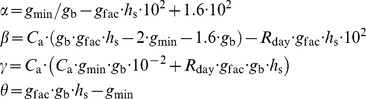	µmol m**^−^** ^2^ s**^−^** ^1^	17	[29]
**Photosynthetic acclimation**				
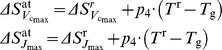 Photosynthetic acclimation to growth temperature	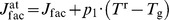	J K**^−^** ^1^ mol**^−^** ^1 ^J K**^−^** ^1^ mol**^−^** ^1^ dimensionless	18a 18b 19	[32]
Photosynthetic acclimation to CO_2_ concentration	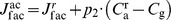 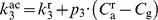	dimensionless µmol g**^−^** ^1 ^N s**^−^** ^1^	20 21	[33]

**Table 2:** Parameters and variables of the photosynthesis - stomatal conductance models.

Symbol	Value	Unit	Description
**Parameters**			
*C*	−0.02	m^2^ ****s****µmol**^−^** ^1^	Slope of the linear relationship between *I*_fac_ and *PPFD* in the range 0–25 µmol m**^−^** ^2^ s**^−^** ^1^
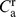	35	Pa	Reference atmospheric CO_2_ partial pressure
*d*	1	µmol CO_2_ m**^−^** ^2^ leaf s**^−^** ^1^	y-intercept of the linear relationship between *I*_fac_ and *PPFD* in the range from 0–25 µmol m**^−^** ^2^ s**^−^** ^1^
*g*_b_	300	mmol m**^−^** ^2^ s**^−^** ^1^	Leaf boundary layer conductance to water vapour
*g*_fac_	13.7	dimensionless	Stomatal sensitivity coefficient
*g*_min_	76.2	mmol m**^−^** ^2^ s**^−^** ^1^	Minimum stomatal conductance to water vapour
*I*_fac_	0.5	dimensionless	Coefficient representing the extent to which *R*_dark_ is inhibited in the light
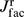		dimensionless	Ratio between *J*_max_ and 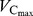 of plant grown at the reference temperature and at the reference CO_2_ partial pressure
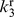		µmol CO_2_ g**^−^** ^1 ^N s**^−^** ^1^	Slope of linear relationship relating *N*_pa_ to 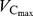 at the reference temperature and at the reference CO_2_ partial pressure
*K*_c_	19.42	Pa	Michaelis-Menten constant for carboxylase activity of Rubisco
*K*_o_	14 300	Pa	Michaelis-Menten constant for oxgenase activity of Rubisco
*O*_i_	21 000	Pa	Internal leaf oxygen concentration
*p*_1_	−0.012	dimensionless	Coefficient representing the extent to which *J*_fac_ is modified by the CO_2_ partial pressure during plant growth
*p*_2_	0.036	dimensionless	Coefficient representing the extent to which *J*_fac_ is modified by the temperature during plant growth
*p*_3_	0.3192	µmol CO_2_ g**^−^** ^1 ^N s**^−^** ^1^	Coefficient representing the effect of CO_2_ partial pressure during plant growth on *k*_3_
*p*_4_	0.94	dimensionless	Coefficient representing the effect of growth temperature on entropy term for *J*_max_ and 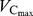
*R*	8.314	J K**^−^** ^1^ mol**^−^** ^1^	Perfect gas constant
*R*_fac_	0.011	dimensionless	Ratio between *R*_dark_ and 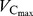 at reference temperature
*SLA*		m^2^ leaf g**^−^** ^1^ DM	Specific leaf area
α	0.05	mol CO_2_ mol**^−^** ^1^ photon	Apparent quantum yield of net photosynthesis at saturating CO_2_
ΔHa_Jmax_	83 608	J mol**^−^** ^1^	Activation energy of *J*_max_
ΔHa_Kc_	65 800	J mol**^−^** ^1^	Activation energy of *K*_c_
ΔHa_Ko_	36 000	J mol**^−^** ^1^	Activation energy of *K*_o_
ΔHa_Rdark_	50 861	J mol**^−^** ^1^	Activation energy of *R*_dark_
ΔHa_Vcmax_	86 529	J mol**^−^** ^1^	Activation energy of 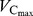
ΔHa_τ_	−28 990	J mol**^−^** ^1^	Activation energy of τ
ΔHd	200 000	J mol**^−^** ^1^	Deactivation energy
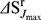	660.42	J K**^−^** ^1^ mol**^−^** ^1^	Entropy term of *J*_max_ for plant grown at reference temperature
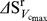	654.24	J K**^−^** ^1^ mol**^−^** ^1^	Entropy term of 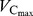 for plant grown at reference temperature
τ	2 838	dimensionless	Rubisco specificity factor at reference temperature
**Input Variables**			
*C*_a_		Pa	CO_2_ partial pressure in the ambient air
*C*_g_		Pa	Atmospheric CO_2_ partial pressure during preceding month of plant growth
*h*_s_		dimensionless	Leaf surface relative humidity
*PPFD*		µmol m**^−^** ^2^ s**^−^** ^1^	Photosynthetic photon flux density
*T*_k_		K	Air temperature. In our analysis *T*_k_ = *T*_g_
*T*_g_		K	Mean air temperature during preceding month of plant growth
*T* ^r^	293.16	K	Reference temperature for metabolic activity
			
**Output variables**			
*A*_n_		µmol m**^−^** ^2^ s**^−^** ^1^	Net photosynthesis
*C*_i_		Pa	Internal CO_2_ partial pressure
*C*_s_		Pa	Leaf surface CO_2_ partial pressure
*g*_s_		mmol m**^−^** ^2^ s**^−^** ^1^	Stomatal conductance to water vapor
*k*_2_		Pa	Intermediate variable synthesizing the Rubisco affinity for CO_2_
*J*		µmol m**^−^** ^2^ s**^−^** ^1^	Light dependence of the rate of electron transport
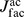		dimensionless	*J*_fac_ acclimated to CO_2_ during plant growth
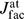		dimensionless	*J*_fac_ acclimated to temperature during plant growth
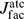		dimensionless	*J*_fac_ acclimated to CO_2_ and to temperature during plant growth
*J*_max_		µmol m**^−^** ^2^ s**^−^** ^1^	Potential rate of RuBP regeneration
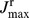		µmol m**^−^** ^2^ s**^−^** ^1^	Potential rate of RuBP regeneration at reference temperature
*k_3_*		µmol CO_2_ g**^−^** ^1 ^N s**^−^** ^1^	Slope of linear relationship relating *N*_pa_ to 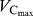
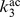		µmol CO_2_ g**^−^** ^1 ^N s**^−^** ^1^	Slope of linear relationship relating *N*_pa_ to 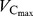 acclimated to CO_2_ during plant growth
*N*_a_		g N m**^−^** ^2^ leaf	Leaf N content per leaf area
*N*_ac_		g N m**^−^** ^2^ leaf	Leaf N content per leaf area when *W*_c_ equals *W*_j_
*N*_pa_		g N m**^−^** ^2^ leaf	Leaf photosynthetic N content per leaf area
*Np*_ac_		g N m**^−^** ^2^ leaf	Leaf photosynthetic N content per leaf area when *W*_c_ equals *W*_j_
*R*_dark_		µmol m**^−^** ^2^ s**^−^** ^1^	Leaf dark respiration rate
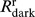		µmol m**^−^** ^2^ s**^−^** ^1^	Leaf dark respiration rate at reference temperature
*R*_day_		µmol m**^−^** ^2^ s**^−^** ^1^	Leaf respiration rate from processes other than photorespiration
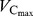		µmol m**^−^** ^2^ s**^−^** ^1^	Maximum carboxylation rate of Rubisco
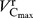		µmol m**^−^** ^2^ s**^−^** ^1^	Maximum carboxylation rate of Rubisco at reference temperature in the absence of any deactivation as a result of high temperature
*W*_c_		µmol m**^−^** ^2^ s**^−^** ^1^	Rubisco-limited photosynthetic rate
*W*_j_		µmol m**^−^** ^2^ s**^−^** ^1^	RuBP regeneration limited photosynthetic rate through electron transport
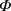		dimensionless	Temperature dependence of *J*_max_ or 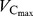
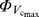		dimensionless	Temperature dependence of 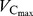
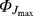		dimensionless	Temperature dependence of *J*_max_
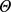		dimensionless	Temperature dependence of *K*_c_, *K*_o_, τ, or *R*_dark_
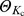		dimensionless	Temperature dependence of *K*_c_
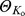		dimensionless	Temperature dependence of *K*_o_
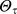		dimensionless	Temperature dependence of τ
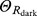		dimensionless	Temperature dependence of *R*_dark_
Γ*		dimensionless	CO_2_ compensation point in the absence of mitochondrial respiration
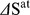		J K**^−^** ^1^ mol**^−^** ^1^	Entropy term acclimated to temperature during plant growth
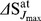		J K**^−^** ^1^ mol**^−^** ^1^	Entropy term of *J*_max_ acclimated to temperature during plant growth
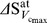		J K**^−^** ^1^ mol**^−^** ^1^	Entropy term of 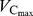 acclimated to temperature during plant growth

Parameter values are derived from Wohlfahrt et al. [3]–[4].

In agreement with the half-life time of Rubisco [26], we assumed that photosynthetic coordination varies with the mean over one month of the environmental conditions during plant growth. We tested the coordination hypothesis: i) by comparing simulated *W*_c_ and *W*_j_ values for the measured *N*_a_, and ii) by comparing simulated (*N*_ac_) and measured (*N*_a_) leaf N contents. Second, thanks to a statistical model, we distinguished the plant species and environmental conditions effects on leaf photosynthetic traits. Third, we tested the implications of our leaf photosynthesis coordination model for net C assimilation (*A*_n_) and for photosynthetic N use efficiency (*PNUE*) by varying plant photosynthetic traits and environmental growth conditions. Based on these results, we discuss the applicability of the coordination hypothesis to predict photosynthetic capacity and N content of sunlit leaves at the ecosystem and global scales.

## Methods

### A Model Coupling Leaf N with CO_2_ and H_2_O Fluxes

Several formulations and parameterizations of the original model by Farquhar et al. [1] have been described. Here, we refer to the formulation and parameterization used by Wohlfahrt et al. [3]. The net rate of C assimilation (*A*_n_, µmol m**^−^** ^2^ s**^−^** ^1^) was limited either by carboxylase activity of Rubisco (*W*_c_, µmolCO_2_ m**^−^** ^2^ s**^−^** ^1^) or by electron flux through the chloroplast photosystems (*W*_j_, µmolCO_2_ m**^−^** ^2^ s**^−^** ^1^) (see Eqn 3–4, 7 in Table 1). Their respective capacity, 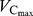 and *J*_max_, scaled with photosynthetic leaf N content (*N*_pa_, gN m**^−^** ^2^) (Eqn 6, 9). The relationship between the intracellular CO_2_ concentration (*C*_i_, Pa) and the stomatal conductance (*g*_s_, mmol m**^−^** ^2^ s**^−^** ^1^) was modeled according to Falge et al. [27] (Eqn 14–17). *g*_s_ can limit *A*_n_ and thereby modify the linearity of the photosynthetic capacities *vs N*_pa_ relationship [28]. An analytical method was used to couple *A*_n_ and *g*_s_, leading to the calculation of *A*_n_ through a system of five equations and five unknowns [29], [30] (Eqn 17). The daytime temperature dependence of 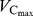 and *J*_max_ was described following Medlyn et al. [31] (Eqn 12). Some studies have shown from a large dataset that the entropy terms of 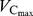 and *J*_max_ acclimate to the mean growth temperature (*T*_g_, K) experienced by leaves over the preceding month [32]. The formalism and parameterization proposed by these authors [32] was used in this study to describe the acclimation of 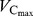 and *J*_max_ to *T*_g_ (Eqn 18–19). Similarly, Ainsworth and Long [33] have shown an acclimation of *A*_n_ to atmospheric CO_2_ concentration during the preceding month (*C*_g_, Pa). This was also taken into account (Eqn 20–21), by modifying the relationship of *Vc_max_* and *J_max_* at standard temperature (*J*_fac_, dimensionless) and the relationship of *Vc_max_* at standard temperature to *N*_pa_ (*k*_3_, µmolCO_2_ g**^−^** ^1^ N s**^−^** ^1^) according to a linear function of the difference between reference (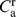) and growth CO_2_ concentrations (*C*_g_).

A sensitivity analysis of the photosynthesis-stomatal conductance model was performed by analyzing the range of parameter variations in literature (Text S1, Table S2) and the sensitivity of the model outputs in response to a ±15% change in parameter values (Text S1, Fig. S2–S3). An index of sensitivity (IOS) was calculated as the ratio of output to parameter changes and was used to discuss on the model uncertainties linked to model calibration.

### Coordinated N Content of Sunlit Leaves

Within leaves, N is partitioned between metabolic and structural pools [24], [25]. The coordinated leaf N content, *N*_ac_ (gN m**^−^** ^2^) is calculated as the sum of structural leaf N and of photosynthetic leaf N (*Np*_ac_, gN m**^−^** ^2^). As leaf structures are highly dependent upon the biomass investment in dry matter (DM) [34], structural leaf N (*f*_ns_, gN g**^−^** ^1^ DM) is expressed per unit DM. *f*_ns_ is assumed constant across species and independent of canopy depth and light intensity. *f*_ns_ value corresponds to the average value reported in the literature for a range of C_3_ species (0.012 gN g**^−^** ^1^ DM, for a review see Lötscher et al. [25]). In contrast, metabolic leaf N associated with leaf photosynthesis is expressed per unit area since both light capture and CO_2_ exchange with atmosphere are intrinsically area-based phenomena [3]. As a key measure of leaf morphology [6], *SLA* links dry matter-based structural N content (*f*_ns_) to area-based photosynthetic N content (*Np*_ac_):
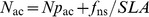
(1)


Under given environmental conditions, *Np*_ac_ is defined as the *N*_pa_ value at which *A*_n_ was co-limited by *W*_c_ and *W*_j_ (Fig. S1). Both 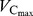
and *J*_max_ are linear functions of *Np*_a_ and, for given environmental conditions, there is a single *Np*_ac_ value for which *W*_c_ equals *W*_j_. At this co-limiting point, *Np*_ac_ equals (see Text S2 Eqn 2a-2d for details):
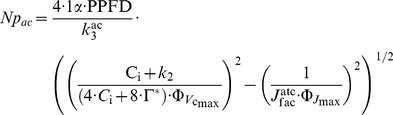
(2)where α (molCO_2_ mol**^−^** ^1^photon) is the apparent quantum yield of *A*_n_ at saturating CO_2_, *PPFD* (µmol m**^−^** ^2^ s**^−^** ^1^) is the photosynthetic photon flux density, 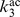
(µmol CO_2_ g**^−^** ^1^N s**^−^** ^1^) is *k*_3_ acclimated to *C*_g_ (Eqn 21), *k*_2_ (Pa) is an intermediate variable synthesizing the Rubisco affinity for CO_2_ (Eqn 5), Γ* (Pa) is the CO_2_ compensation point in the absence of mitochondrial respiration, 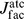 is *J*_fac_ acclimated to *C*_g_ and *T*_g_ (CO_2_ air concentration and temperature during preceding month of plant growth, Eqn 19–20), and 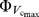 and 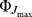
(dimensionless) are the response functions of 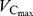 and *J*_max_ to temperature (Eqn 12). Overall, *Np*_ac_ integrates the sensitivity of photosynthetic machinery to *T*_g_, *PPFD*, *C*_i_ and *h*_s_.

### Dataset

A dataset was assembled from measurements and literature to associate leaf photosynthetic traits of mature sunlit leaves with environmental growth conditions (Dataset SI4). 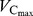 and *J*_max_ at reference temperature (*T* ^r^  = 20°C), *N*_a_, *SLA*, as well as *T*_g_, *PPFD*, *h*_s_ and *C*_g_ during the month preceding leaf measurements were included. 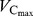 and *J*_max_ values were standardized using a consistent formulation and parameterization of Γ* and the Michaelis-Menten constants for carboxylase (*K*_c_, Pa) and oxygenase (*K*_o_, Pa) Rubisco activity [32], [35].

The dataset has 293 entries from 31 C_3_ plant species covering six plant functional types (PFTs): temperate broadleaved and coniferous evergreen trees (PFT1), temperate broadleaved deciduous trees (PFT2), deciduous shrubs and herbs (PFT3), perennial C_3_ grasses and forbs (PFT4), C_3_ crops (wheat, PFT5) and N-fixing trees (PFT6). The final dataset covers a wide range of plant growth conditions: *T*_g_ (ranging from 7.1 to 21.0°C), *PPFD* (500 to 1170 µmol m**^−^** ^2^ s**^−^** ^1^), *h*_s_ (0.51 to 0.89) and *C*_g_ (36 and 60 Pa). However, data corresponding to severe drought and/or to very low N availability during growth were excluded from the dataset. Four categories of inorganic N availability (low, medium, high and very high), two categories of soil moisture and of atmospheric CO_2_ concentration (ambient and elevated) and six categories of experimental set-up (climate chamber, sunlit climate chamber, botanical garden, natural vegetation, free air CO_2_ enrichment (FACE) and open top chambers) were defined. The dataset has been made available via the TRY initiative on plant traits [36].

### Data Analysis

#### Coordinated *W*_c_ and *W*_j_

The basic assumption of the coordination hypothesis is that under the environmental conditions to which a leaf is adapted, RuBP carboxylation equals RuBP regeneration (*W*_c_  =  *W*_j_). Here we tested this for the average daily plant growth conditions (excluding night values) during the last month preceding photosynthesis measurements. We used four environmental variables (*C*_g_, *PPFD*, *T*_g_ and *h*_s_) corresponding to the average plant growth conditions as model input, and 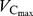 and *J*_max_ derived from separate photosynthesis measurements on the same plants. A single set of values was used for all other 33 model parameters and was originated from Wohlfahrt’s calibration (Table 2) [3], [4]. *W*_c_ and *W*_j_, both predicted for the average plant growth conditions for each observation (n  = 293), were compared by least square linear regression. Regression residuals were analyzed using a general linear model (GLM) with *T*_g_, *h*_s_, *C*_g_ and with PFTs and N categories. PFTs and N levels were compared by the post ANOVA Tukey’s HSD method.

#### Prediction of the coordinated leaf N content

*N*_ac_ was calculated for each observation (n  = 293) using four environmental variables (*C*_g_, *PPFD*, *T*_g_ and *h*_s_) corresponding to the growth conditions of the past month and three leaf traits (*k*_3_, *J*_fac_ and *SLA*). *k*_3_ is calculated as the ratio between 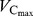 and *Np*_a_, while *J*_fac_ is calculated as the ratio between *J*_max_ and 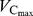. The prediction of *N*_ac_ was evaluated by the relative root mean squared error (RRMSE), which is the relative average of the squared differences between predicted and observed values [37]. RRMSE values lower than 0.2 indicates here acceptable errors. Systematic (RRMSE_S_) and unsystematic (RRMSE_U_) errors [37] specified the error source of RRMSE (Eq. I).
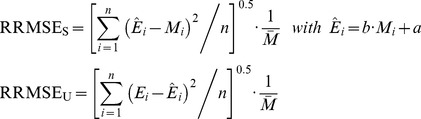
(I)where *E*_i_ and *M*_i_ are the predicted and measured values of the observation *i*, 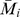 is the average of *M*_i_ and 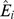 is an estimate of *E*_i_ deriving from the linear regression between *E*_i_ and *M*_i._

#### Dependence of leaf photosynthetic parameters on plant functional type (PFT)

ANOVA followed by LSD method for mean comparison tests, were used to analyze the role of PFT for the estimation of leaf photosynthetic traits used in the test of the coordination hypothesis (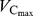, *J*_max_, *k*_3_, *J*_fac_ and *SLA*). In order to test if the calibration of leaf photosynthetic traits can be simplified to obtain a unique value or a value by PFT, we estimated independent values of *k*_3_, *J*_fac_ and *SLA* traits minimizing the squared differences between *N*_a_ and *N*_ac_ (Newton’s optimization method). Mean and optimized values per PFT were then compared by linear regressions. The calibration of leaf traits by species was not tested since the number of observations per species was too variable in our dataset.

#### Dependence of leaf photosynthetic parameters on environmental growth conditions

Multiple regression models were used to analyze the effects of environmental growth conditions (*T*_g_, *PPFD*, *h*_s_ and *C*_g_, N and soil moisture categories) on leaf traits (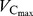, *J*_max_, *k*_3_, *J*_fac_ and *SLA*). For regression models of *k*_3_ and *J*_fac_, the values of dependent variables were log-transformed and all residuals followed a normal distribution.

We tested if the prediction of leaf photosynthetic traits by environmental growth conditions was robust and validated likewise the coordination hypothesis. We conducted bootstrap analyses to predict *W*_c_ and *W*_j_ as a function of 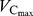 and *J*_max_ estimated by an independent regression model and environmental growth conditions. In the same way, bootstrap analyses were conducted to predict *N*_ac_ as a function of estimated *k*_3_ and *J*_fac_. To do so, two-thirds of the 293 observations were randomly used to parameterize the multiple regression models (20 random sets, Tables S3–S4). These models were used to predict the leaf photosynthetic parameters 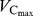, *J*_max_, *k*_3_ and *J*_fac_ of the remaining observations from their environmental growth conditions. As *SLA* was not predictable from environmental growth conditions *(*see in result the low coefficient of determination in *SLA* regression model), experimental specific values were used. Finally, *W*_c_, *W*_j_ and *N*_ac_ were calculated and the coordination hypothesis was evaluated again (Tables S5–S6).

We also attempted to falsify the testable hypothesis (*W*_c_  =  *W*_j_ and *N*_a_  =  *N*_ac_) provided by the photosynthetic coordination hypothesis. To this end, we randomized environmental growth conditions among observations (permutation test) and tested the alternative hypothesis significant differences between *W*_c_ and *W*_j_ and between *N*_a_ and *N*_ac_.

#### Prediction from our leaf photosynthesis coordination model

The implications of the coordination hypothesis for *N*_ac_, *A*_n_ and *PNUE* were tested by varying: i) the values of the leaf parameters *k*_3_ and *J*_fac_ under mean environmental growth conditions (*PPFD* = 666 µmol m**^−^** ^2^ s**^−^** ^1^, *T*_g_ = 16.9°C, *h*_s_ = 0.74); ii) the values of the environmental growth parameters *T*_g_ and *PPFD* assuming mean leaf photosynthetic parameter values (*k*_3_ = 59.1 µmol g**^−^** ^1^ *Np*_a_ s**^−^** ^1^; *J*_fac_ = 2.45; *SLA* = 17.7 m^2^ kg**^−^** ^1^ DM).

All statistical tests were performed using Statgraphics Plus (v. 4.1, Manugistics, USA).

## Results

### Leaf Photosynthesis Shows Co-limitation Under Mean Growth Conditions

**Figure 1:**
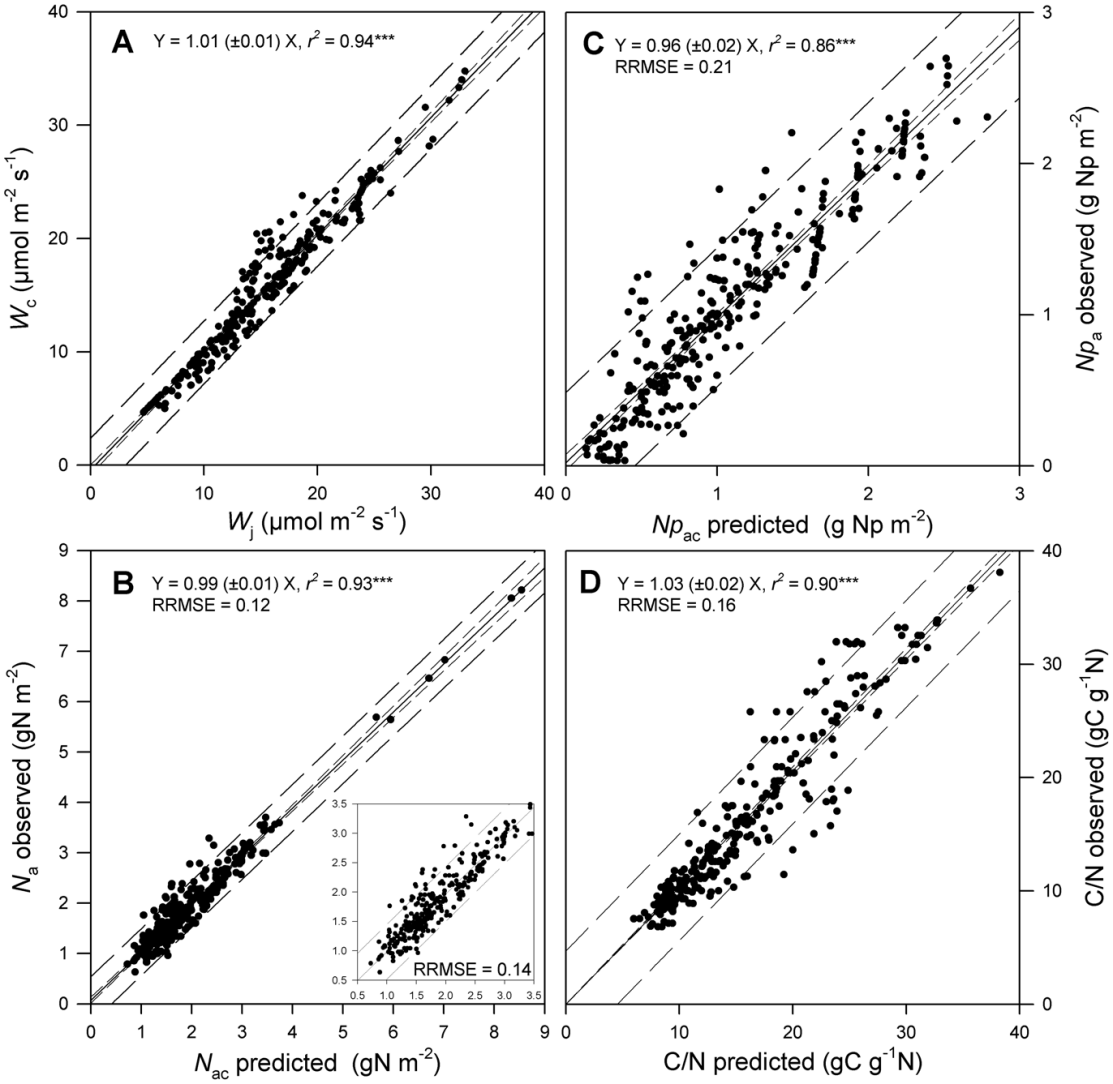
Tests of the coordination hypothesis using experimental values of leaf photosynthetic traits (*Vc*_max_, *J*_max_, *J*_fac_, *k*_3_ and *SLA*). A) Relationship between the predicted rates of RuBP carboxylation/oxygenation (*W*_c_) and RuBP regeneration (*W*_j_) under plant growth conditions. B) Relationship between predicted (*N*_ac_) and observed (*N*_a_) leaf N content. *N*_a_ was calculated as the sum of the leaf photosynthetic and structural N contents. Leaf photosynthetic N content was predicted using Eqn 2 with the species-specific parameters *k*_3_ and *J*_fac_. C) Relationship between predicted (*Np*_ac_) and observed (*Np*_a_) photosynthetic leaf N content. D) Relationship between predicted and observed leaf C/N ratio. A common leaf structural N content was used (*fns*  = 0.012 gN g**^−^** ^1^ DM). Solid lines are the regressions. Short-dashed and long-dashed lines indicate the confidence (at 95%) and prediction intervals, respectively. The insert in Fig. 1B shows the same relationship without the very high observed *N*_a_ values for the PFT1. ***, *P*<0.001.

We assessed the level of photosynthetic co-limitation by comparing dark (*W*_c_) to light-driven (*W*_j_) biochemical processes under growth conditions experienced by the leaves in the month prior to observations. *W*_c_ strongly correlated with *W*_j_ (Fig. 1A, n = 293, *P*<0.001, intercept not significantly different from zero) across species and growth environments (characterized by *T*_g_, *PPFD*, *h*_s_ and *C*_g_). An ANOVA on the regression residuals revealed a significant PFT effect (*d.f.* = 5, 283; *P*<0.001; data not shown). The calculated *W*_c_/*W*_j_ ratio was not significantly different from one (*t*-test at *P*<0.05, n = 293). This ratio varied neither with species parameters, nor with environmental growth conditions.

### Predicted Coordinated Leaf N Content (*N*_ac_) Matches Observed Leaf N Content (*N*_a_)

Overall, predicted and observed *N*_a_ values were closely correlated with a slope not significantly different from one and an intercept not significantly different from zero (Fig. 1B, n = 293, *P*<0.001, RRMSE  = 0.12). The breakdown of RRMSE into unsystematic and systematic error terms showed that the prediction error was mostly unsystematic and therefore associated to data and not to a systematic model error (RRMSEs  = 0.012; RRMSEu  = 0.108). An ANOVA on the residuals of the prediction showed weak but significant effects of PFTs, *T*_g_ and *h*_s_ (*d.f.*  = 5, 1, 1, respectively; *P*<0.01; data not shown).

As *f*_ns_ was assumed constant across species [25], we calculated *N*_pa_ and *N*_pac_ by subtracting the ratio *f*_ns_/*SLA* to *N*_a_ and *N*_ac_, respectively. Similarly, predicted and observed *Np*_a_ values were closely correlated (Fig. 1C, n = 293, *P*<0.001, RRMSE  = 0.21).

As carbon content in leaves was assumed to be approximately constant, we calculated a C/N ratio by dividing *N*_a_ and *N*_ac_ by the ratio between a common carbon content (*fcs*  = 0.45 gC g**^−^** ^1^ DM; [36], [38]) and *SLA*. Predicted C/N matched significantly the calculated C/N, observed across environmental conditions and across species and PFTs (Fig. 1D).

### Dependency of Leaf Parameters on Plant Functional Type

In the dataset (Table S1), the parameters used to calculate leaf photosynthesis and stomatal conductance were *SLA*, *J*_fac_, *k*_3_, calculated from 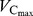, *J*_max_ and leaf N measurements (Eqn 12, 15). At *T* ^r^, 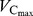 and *J*_max_ varied between 4–141 µmol m**^−^** ^2^ s**^−^** ^1^ and 8–213 µmol m**^−^** ^2^ s**^−^** ^1^, respectively. *k*_3_ varied from 4.6 to 350 µmol g**^−^** ^1^N s**^−^** ^1^ while *J*_fac_ values were very constrained from 1.69 to 3.71, as already observed [2]. Finally, *SLA* varied from 1.5 to 43.2 m^2^ kg**^−^** ^1^ DM. All photosynthetic traits showed significant dependency to PFT (*P*<0.001) but with different determination coefficient (*r* ^2^ = 0.66, 0.64, 0.24, 0.47 and 0.40 for 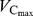, *J*_max_, *k*_3_, *J*_fac_ and *SLA*, respectively). Post-ANOVA LSD tests showed that the discrimination among the PFTs was more effective for *J*_fac_, *J*_max_ and *SLA* separating significantly four groups among the six PFTs (Table S7) and was much weaker for *k*_3_ and 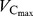 (two groups were significantly distinguished).

*k*_3_, *J*_fac_ and *SLA* can be optimized to a value which minimizes the squared differences between *N*_a_ and *N*_ac_ (Table 3A). When *k*_3_ was optimized by PFT, *N*_a_ was accurately predicted (slope  = 0.96, *r* ^2^ = 0.73, RRMSE  = 0.23). When a single value was used for the whole dataset, *N*_a_ prediction was not satisfactory. The optimization by PFT of *J*_fac_ led to a strong prediction of *N*_a_ (slope not different from one, *r* ^2^ = 0.79, RRMSE  = 0.23). When a single value was used for the entire dataset (*J*_fac_  = 2.11), the prediction of *N*_a_ was less accurate but the slope of the relationship between *W*_c_ and *W*_j_ remained close to one. Finally, the optimisation of *SLA* by PFT or to a single value for the entire dataset strongly reduced the accuracy of *N*_a_ prediction. Optimization of the *k*_3_ and *J*_fac_ parameters showed that *N*_a_ can be acceptably predicted when their values are defined by PFT. For all traits, average values by PFT and optimized values by PFT displayed significant linear relationships (Table 3B).

**Table 3:** Estimates of the optimized value (for the entire dataset and by PFT) of leaf photosynthetic traits (*J*_fac_, *k*_3_ and *SLA*).

A)	Optimized value				*W*_c_/*W*_j_			*N*_a_/*N*_ac_			
Parameter					Slope		*r* ^2^	Slope	*r* ^2^		RRMSE
*k*_3_											
All	48.3				1.15±0.02		0.78	0.94±0.02	0.64		0.28
PFT	45.2; 37.1; 54.0; 79.4; 46.2; 24.2				1.08±0.02		0.88	0.96±0.02	0.73		0.23
						
*J*_fac_											
All	2.11				1.06±0.02		0.89	0.97±0.02	0.68		0.31
PFT	2.11; 2.11; 2.59; 1.70; 2.33; 3.10				1.04±0.02		0.92	1.02±0.02	0.79		0.23
						
*SLA*											
All	17.7				1.02±0.02		0.92.	0.88±0.02	0.43		0.44
PFT	8.1; 13.7; 18.2; 20.0; 18.3; 13.4				1.02±0.02		0.92.	0.96±0.02	0.48		0.37
						
*k*_3_ and *J*_fac_											
All	*k*_3_ = 48.3; *J*_fac_ = 2.11				1.18±0.02		0.79	0.89±0.02	0.68		0.33
PFT	*k*_3_ = 45.2; 37.1; 54.0; 79.4; 46.2; 24.2 *J*_fac_ = 2.11; 2.11; 2.59; 1.70; 2.33; 3.10				1.06±0.02		0.88	0.96±0.02	0.74		0.26
**B)**		***k*** **_3_**		***J*** **_fac_**				***SLA***			
**PFT**		**Mean**	**Optimized**	**Mean**		**Optimized**		**Mean**		**Optimized**	
PFT1		65.0	45.2	2.23		2.11		11.1		8.1	
PFT2		46.6	37.1	2.32		2.11		13.1		13.7	
PFT3		90.1	54.0	2.53		2.59		21.4		18.2	
PFT4		86.1	79.4	2.04		1.7		22.0		20.0	
PFT5		44.9	46.2	2.69		2.33		18.3		18.3	
PFT6		38.1	24.2	2.50		3.1		20.3		13.4	
Correlation		*r* ^2^ = 0.68	*P*<0.001	*r* ^2^ = 0.49		*P*<0.001		*r* ^2^ = 0.68		*P*<0.001	

The squared difference between measured *N*_a_ and predicted *N*_ac_ values were minimized by Newton’s method. A) The optimization was done with one trait at a time without changing the values of the two other traits. The optimized values are ordered by PFT (i.e. the first value corresponds to PFT1). B) The optimized values by PFT were compared to mean per PFT in the dataset by using a linear regression model. Abbreviations: PFT1, temperate broadleaved and coniferous evergreen trees; PFT2, temperate broadleaved deciduous trees; PFT3, deciduous shrubs and herbs; PFT4, perennial C_3_ grasses and forbs; PFT5, C_3_ crops (wheat); PFT6, N-fixing trees.

### Dependency of Leaf Parameters to Environmental Growth Conditions

All leaf photosynthetic parameters could be predicted from environmental growth conditions (Table 4). However, *SLA* was poorly correlated with environmental conditions (*r* ^2^ = 0.15). *J*_max_ was reasonably well predicted by environment (*r* ^2^ = 0.64, *P*<0.001). It was predominantly affected by the N level experienced by plants during growth (36% of explained variance), with a high N level leading to higher *J*_max_ values. *J*_max_ was then positively affected by *PPFD* (7%), *h*_s_ (13%), and *PPFD* times *T*_g_ (5%) and was negatively affected by soil moisture level (12%), *T*_g_ (9%), and *PPFD* times *h*_s_ (18%). 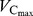, which was significantly predicted from environmental condition during growth (*r* ^2^ = 0.66, *P*<0.001), was mainly affected by *T*_g_ (33%, negatively), N level (25%, positively) and soil moisture level (15%, negatively). Then, 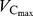 was positively affected by *PPFD* (8%) and *h*_s_ (5%) and was negatively affected by CO_2_ level (5%) and *PPFD* times *h*_s_ (8%).

**Table 4:** Effects of environmental conditions on the leaf photosynthetic traits: 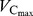 *J*_max_, *J*_fac_, *k*_3_ and *SLA*.

A)		*J*_max_		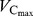		log *J*_fac_		log *k*_3_		*SLA*	
Factors	d.f.	Variance	*P*-value	Variance	*P*-value	Variance	*P*-value	Variance	*P*-value	Variance	*P*-value
CO_2_ level	1	.	ns	4.6	<0.01	27.0	<0.001	.	ns	.	ns
N level	3	35.5	<0.001	24.5	<0.001	9.8	<0.05	65.1	<0.001	7.3	<0.05
H_2_O level	1	12.2	<0.001	15.3	<0.001	8.1	<0.01	.	ns	3.1	<0.01
*PPFD*	1	6.6	<0.01	8.9	<0.001	5.7	<0.05	2.1	<0.05	0.1	<0.01
*T*_g_	1	9.5	<0.01	33.1	<0.001	.	ns	25.3	<0.001	77.9	<0.001
*h*_s_	1	12.7	<0.001	5.4	<0.01	19.2	<0.001	4.5	<0.01	1.8	<0.05
PPFD*T_g_	1	5.4	<0.05	.	ns	6.0	<0.05	.	ns	.	ns
PPFD*h_s_	1	18.1	<0.001	8.2	<0.001	24.2	<0.001	3.0	<0.05	9.7	<0.05
Overall	293	*r* ^2^ = 0.64	<0.001	*r* ^2^ = 0.66	<0.001	*r* ^2^ = 0.51	<0.001	*r* ^2^ = 0.44	<0.001	*r* ^2^ = 0.15	<0.01

The factors are environmental growth conditions: radiation (*PPFD*), temperature (*T*_g_), relative humidity (*h*_s_), air CO_2_ concentration (CO_2_ level), soil N availability (N level) and soil moisture (H_2_O level). A) Degree of freedom (*d.f.*), variance explained (%), statistical significance and sign (positive or negative) of interactions with continuous variables. B) Coefficients estimate of ANOVA model. All variable values were analyzed at a reference temperature of 20°C. Residuals of analysis followed a normal distribution without transformation for 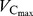 and *J*_max_, and with log-transformation for *J*_fac_ and *k*_3_. We only included in the ANOVA model the interactions that were significant.

*J*_fac_ was significantly predicted from environment (*r* ^2^ = 0.51, *P*<0.001) and the variance was shared between CO_2_ level (27%, positively), *h*_s_ (19%, positively), and *PPFD* times *h*_s_ (24%, negatively). Note that *J*_fac_ increased with CO_2_ concentration as reviewed by Ainsworth and Long [33]. The remaining variance was positively explained by *PPFD* (6%) and *PPFD* times *T*_g_ (6%) and negatively explained by N and moisture levels (10 and 8%, respectively). *k_3_* was significantly predicted (*r* ^2^ = 0.44, *P*<0.001) and the variance was predominantly explained by N level (65%), with higher *k*_3_ at lower N availability level, as also reviewed by Ainsworth and Long [33]. The temperature experienced by leaves during the preceding month was also an important driver of *k*_3_ (25%), with lower *k*_3_ at higher temperature. The remaining variance was positively explained by *PPFD* (2%) and *h*_s_ (4%) and negatively explained by *PPFD* times *h*_s_ (3%).

**Figure 2:**
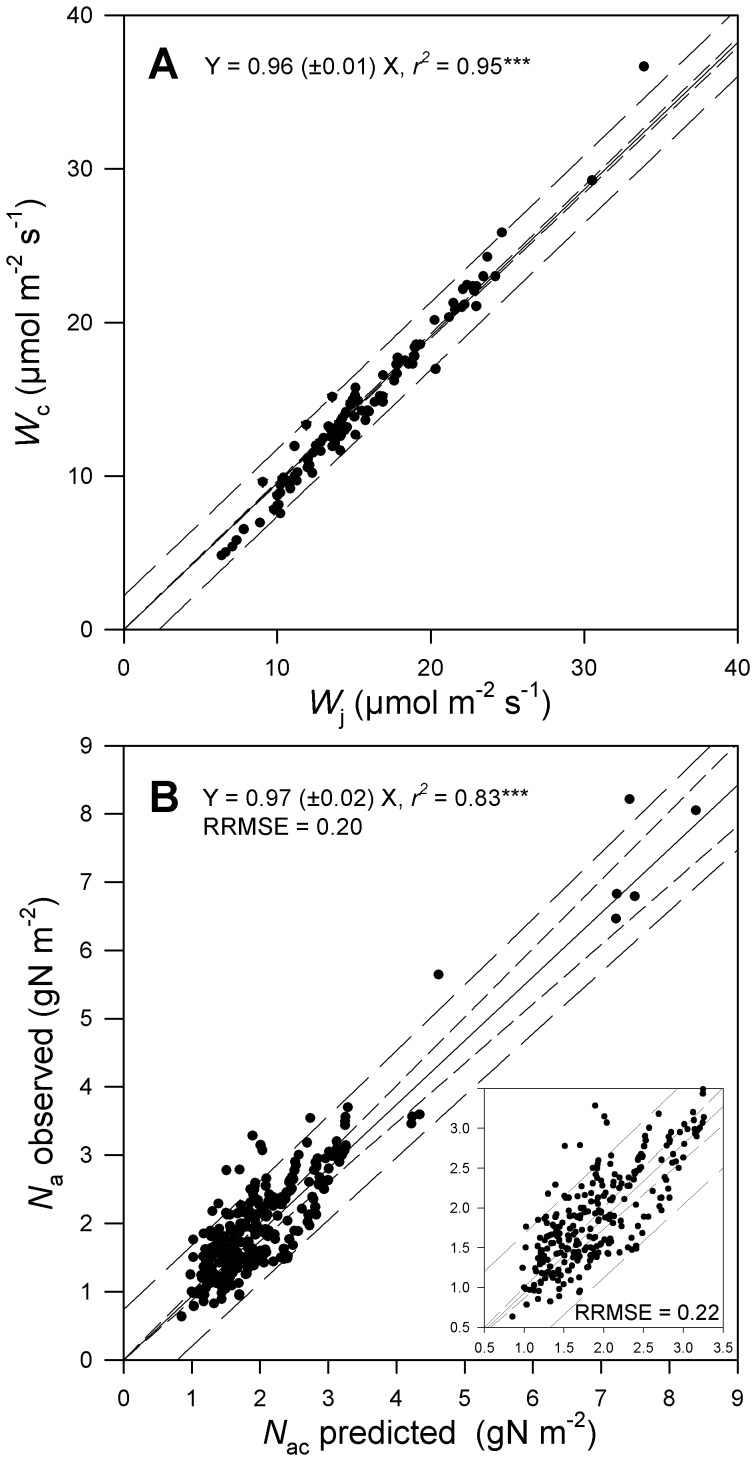
Tests of the coordination hypothesis using values of leaf photosynthetic traits predicted from environmental growth conditions. A) Relationship between the predicted rates of RuBP carboxylation/oxygenation (*W*_c_) and RuBP regeneration (*W*_j_) under plant growth conditions. B) Relationship between predicted (*N*_ac_) and observed (*N*_a_) leaf N content. The insert in Fig. 2B shows the same relationship without the very high observed *N*_a_ values for the PFT1. Symbols are as for Fig. 1.

Once the multiple regression models were established for each leaf photosynthetic parameter, we tested by bootstrap analysis if their prediction was robust enough to satisfy the coordination hypothesis. All random datasets generated by bootstrap (n = 220) gave significant regression models (Tables S5–S6). The parameters values of these regression models were used with the remainder of the data (n = 293–220 = 70) to predict leaf photosynthetic parameters values. Photosynthetic parameters values were then used to predict *W*_c_, *W*_j_ and *N*_ac_. We found that *W*_c_ matched *W*_j_ (Fig. 2A) and *N*_ac_ matched *N*_a_ (Fig. 2B, RRMSE  = 0.2), whatever the random dataset to which it was applied (Tables S5–S6).

In an attempt to falsify the leaf photosynthesis coordination hypothesis, we have randomized environmental growth conditions among observations. This randomization resulted in a strong mismatch between *W*_c_ and *W*_j_ (RRMSE  = 0.76; slope  = 0.60±0.33; *r* ^2^ = 13%) as well as between *N*_a_ and *N*_ac_ (RRMSE  = 0.72; slope  = 0.80±0.40; *r* ^2^ = 17%).

### Prediction from Our Leaf Photosynthesis Coordination Model

**Figure 3:**
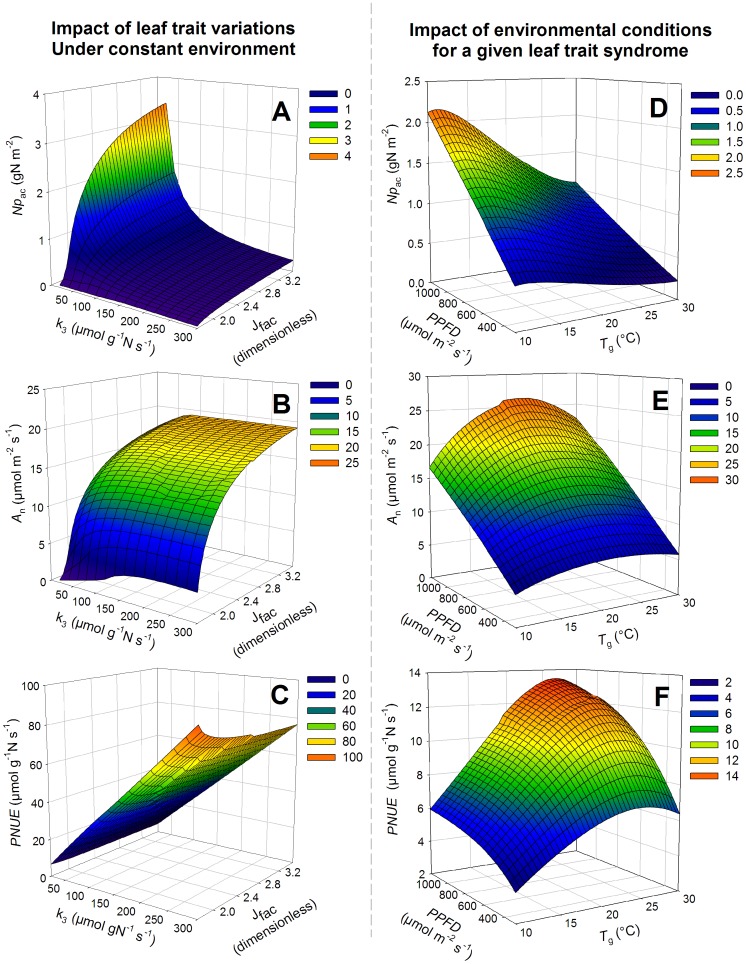
Relationships between simulated photosynthetic leaf N content (*Np*_ac_) (A), net photosynthesis (*A*_n_) (B) and photosynthetic N use efficiency (*PNUE*) (C) and the photosynthetic traits *k*_3_ and *J*_fac_ under standard mean environmental conditions (*PPFD*  = 666 µmol m^−2^ s^−1^, *T*_g_ = 16.9°C, *h*_s_ = 0.74). *k*_3_ is the ratio between 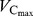 and *Np*_a_. *J*_fac_ is the ratio between *J*_max_ and 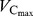. A mesh of *k*_3_ values varying between 10 and 300 µmol g^−1^ N s^−1^ with 20 steps and of *J*_fac_ values varying between 1.75 and 3.5 with 0.05 steps was used. Figures D–E–F, relationships between (*Np*_ac_) (D), net photosynthesis (*A*_n_) (E) and photosynthetic N use efficiency (*PNUE*) (F) and the radiation (*PPFD*) and temperature (*T*_g_) conditions during growth. Averages over the dataset of leaf photosynthetic parameters (*k*_3_, *J*_fac_ and *SLA*) are used (*k*_3_ = 59.1 µmol g^−1^ *N*_pa_ s^−1^, *J*_fac_ = 2.45, *SLA*  = 17.7 m^2^ kg^−1^ DM). The mesh for temperature is 0.5°C between 10 and 30°C and the mesh for radiation is 50 µmol m^−2^ s^−1^ between 300 and 1200 µmol m^−2^ s^−1^. The values of *h*_s_ and *T*_g_ were fixed at 0.8 and 20°C, respectively. *A*_n_ was calculated with the coordinated leaf protein content and *PNUE* was calculated as the ratio between *A*_n_ and *Np*_ac_.

Under standard environmental conditions, *Np*_ac_ varied significantly with *k*_3_ and *J*_fac_ (Fig. 3A). *Np*_ac_ decreased with increasing *k*_3_ (Fig. 3A), which imposed a strong constraint on this physiological trait. For a given leaf *Np*_ac_, high values of *k*_3_ did not affect *A*_n_ (Fig. 3B), but *PNUE* increased linearly with *k*_3_ (Fig. 3C). For a given *k*_3_ value, both *Np*_ac_ (Fig. 3A) and *A*_n_ (Fig. 3B) displayed saturating responses to increasing *J*_fac_. As a consequence, *PNUE* was little affected by *J*_fac_ (Fig. 3C). In our model (Eqn 1), *SLA* and *f*_ns_ affected *N*_ac_, but did not affect *Np*_ac_ and consequently *A*_n_ and *PNUE*. Since *SLA* displayed a higher degree of variation, the leaf structural content per unit area and consequently the leaf N content were strongly dependent on *SLA*. Thus, the leaf structural N content per unit area and the leaf N content followed an inverse relationship as *SLA* increased.

When using overall dataset means of the leaf photosynthetic traits, *Np*_ac_ varied significantly with radiation and temperature (Fig. 3D). *Np*_ac_ increased linearly with *PPFD* and decreased with *Tg* according to a logistic curve (Fig. 3D, Fig. S2). For a given *Np*_ac_, temperature affected *A*_n_ according to a quadratic curve with an optimal *Tg* around 20°C although *PPFD* affected linearly *A*_n_ (Fig. 3E). As a consequence, *PNUE* was affected by *Tg* according to a peak curve with an optimal *Tg* at 25°C and was positively affected by *PPFD* according to a logarithmic curve (Fig. 3F).

## Discussion

### A Successful Test of the Coordination Hypothesis of Leaf Photosynthesis

The coordination hypothesis provides a testable analytical solution to predict both photosynthetic capacity and area-based leaf N content and, hence, to couple photosynthetic C gain and leaf N investment. With the large dataset used in this study, we could not falsify this testable hypothesis. Therefore, our results strongly support the validity of the leaf photosynthetic coordination hypothesis across a wide range of C_3_ plant species and of environmental conditions.

Our coordination model linking leaf photosynthesis, stomata conductance and nitrogen investment has a total of 33 parameters. Only four parameters are directly related to a coordinated investment of leaf N into carboxylation capacity (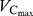; RuBP carboxylation; Rubisco) and electron transport capacity (*J*_max_, RuBP regeneration; light harvesting): *J*_fac_, the ratio of *J*_max_ to 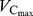 determines the photosynthetic capacity; and *k_3_*, the ratio of 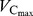 to leaf photosynthetic N content (*Np*_ac_) determines the fraction of metabolic leaf N invested in photosynthesis. The ratio of *f*_ns_ to *SLA* determines the fraction of non-metabolic N per unit total leaf N.

Photosynthetic parameter values vary to a considerable extent across species and environmental conditions in agreement with previous studies [2], [3], [39]. For instance, Wullschleger [2] reported that, when expressed at a reference temperature of 20°C, 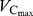 varies in the range 5–142 (µmol m**^−^** ^2^ s**^−^** ^1^); *J*_max_ in the range 11–251 (µmol m**^−^** ^2^ s**^−^** ^1^) and *J*_fac_ in the range 0.9–3.8 (dimensionless). Despite similar large differences in our dataset in parameter values across species and environmental conditions, our photosynthetic coordination model accounts for 93% of the total variance in *N*_a_. Moreover, the model has a low systematic RRMSE with no systematic bias. The statistical validity of this model supports the conclusion that sunlit mature leaves of C_3_ plants tend to achieve photosynthetic coordination in a wide range of both optimal and sub-optimal environmental conditions.

Along the vertical profile of C_3_ plant canopies, an empirical scaling law between area based leaf N content and transmitted *PPFD* has often been reported [15], [17], [40], [41] and has been determined as the predominant factor of N decline relative to others like leaf age or N demand [12], [40], [41]. Various hypotheses have been put forward to explain this observation [11], [22], [42], [43]. Our model of the coordination hypothesis matches this scaling law, since *Np*_ac_ scales with radiation (*PPFD*) along the vertical canopy profile (Eqn. 2). Air temperature (*T*_g_), relative air humidity (*h*_s_) and ambient CO_2_ concentration (*C*_a_) also vary with depth within the canopy. At a given *PPFD*, higher *h*_s_ and lower *T*_g_ at depth would reduce *Np*_ac_, while a lower *C*_a_ would increase it. For some crop species like wheat, N limitation has been reported to accelerate the decline in *N*_a_ with *PPFD* [25], [40], [41], which may indicate preferential N allocation to leaves in full light, resulting in preferential photosynthetic coordination of these leaves despite N limitation.

Variations in photosynthetic N protein contents (*Np*_ac_) appear to be an overwhelming determinant of *N*_a_. In contrast, structural leaf N (*f*_ns_) values varied only within a narrow range [38], when they were optimized by species or by PFT (from 0.0107 to 0.0135 gN g**^−^** ^1^ DM for wheat and N-fixing trees, respectively, corresponding to 0.61 and 0.78 gN m**^−^** ^2^ leaf when *SLA* is set to 17.6 m^2^ kg**^−^** ^1^ DM, dataset mean). Although optimized *f*_ns_ values showed little variations on a leaf dry mass basis, it accounted for 15–50% of *N*_a_ (gN m**^−^** ^2^), across all species in the dataset due to the strong variation in *SLA* across all species. Structural N is found in cell walls (1.6–9.5% of leaf N in *Polygonum cupsidatum* and 40–60% for sclerophyllous tree, shrub and vine species, [34], [44]) and in nucleic acids (10–15%, [45]). In addition, other non-photosynthetic nitrogenous compounds (*e.g.* cytosolic proteins, amino acids, ribosomes and mitochondria) contribute to the structural leaf N pool [46]. Several experimental studies have attempted to estimate *f*_ns_, reporting values between 0.0101 and 0.0136 gN g**^−^** ^1^ DM for a range of herbaceous C_3_ species [16]. These *f*_ns_ values are in the same range as those found for dead leaves after N resorption at senescence [47]. Structural N would therefore not be redistributed by this process [48].

### Determinism of Leaf N Content Variation

Genetic and environmental factors have long been recognized to interact in determining the *A*_max_ vs. leaf N relationship [5]. Our study provides a means for disentangling: i) the direct environmental effects on leaf photosynthetic N content (*Np*_ac_); ii) the role of photosynthetic parameters for *Np*_ac_ in a given environment; and iii) the response of photosynthetic parameters i.e. the plant acclimation to plant growth environment.

First, for a given set of plant parameters, positive effects of radiation and negative effects of air temperature, air relative humidity and CO_2_ concentration on *Np*_ac_ are predicted by Eqn 2 (Fig. 3D–F). These results are in accordance with the prediction by Farquhar et al’s canopy photosynthesis model [49], which links stomatal control with leaf area and leaf N content by optimizing both water and nitrogen use efficiency and predicts an increase of leaf N content and 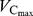 with mean radiation increase [24], [50] and mean annual rainfall [49], [51]. According to the coordination hypothesis, changes in *Np*_ac_ affect both biochemical photosynthesis capacities, 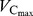
and *J*_max_. Indeed, seasonal variations in 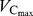 and *J*_max_ have been observed for a number of plant species [52], [53] and were related to changes in Rubisco and cytochrome-f contents in *Polygonum cuspidatum* [54]. Including photosynthetic capacity (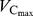 and *A*_max_) and its relationship to leaf N content in terrestrial biosphere models resulted in substantial changes in gross primary productivity with latitude [7]. Coupled environmental variations in *PPFD*, *T*_K_, *h*_s_ and *C*_a_ simultaneously affect *Np*_ac_ throughout time, which has major implications for gross primary productivity and *PNUE* of a given species or genotype.

Second, the coordination hypothesis implies that under a given environment, *N*_a_ tends toward a unique coordinated *N*_ac_ value (Eqn 2). As shown by the analysis of model sensitivity to parameters and input variables (Text S1, Fig. S3), *k*_3_ and *J*_fac_ are among the most important determinants of *N_ac_* value. Assuming a single average value of *k*_3_ and of *J*_fac_ for all species in the dataset would increase *N*_a_ RRMSE by 50% (Table 3A). However, using a single *J*_fac_ value by PFT with species-specific *k*_3_ and *SLA* values provided a strong accuracy for *N*_a_ prediction. This result is consistent with the strong linear relationship between 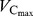 and *J*_max_ reported by Wullschleger [2] among 109 species, which probably indicates a phylogenetic constraint for *J*_fac_. Under given environmental conditions, our results show that there is no single combination of *k*_3_ and *J*_fac_ that can maximize both *A*_n_ and *PNUE* (Fig. 3A–C). Therefore, variable combinations of these photosynthetic traits could be equally relevant. This relative independency of *k*_3_ and *J*_fac_ suggests that these functional traits (*sensu* [55]) correspond to possibly overlooked axes of differentiation among C_3_ plant species. *k*_3_, which modulates the N investment at a given *A*_n_, could be related to a plant strategy of nutrients conservation [56]. *J*_fac_, which increases *A*_n_ for a given *k_3_*, could be related to a plant strategy of nutrients exploitation. However, the lack of correlation between these two photosynthetic traits and *SLA*, which is a key morphological trait separating exploitative and conservative species strategies for nutrient use [56], suggests that these physiological traits form a secondary axis of differentiation across C_3_ species.

Third, some environmental growth conditions such as *PPFD*, *T*_g_, *h*_s_, *C*_a_ and N availability had significant effects on *k*_3_ and *J*_fac_. The increase in *k*_3_ at low N availability tends to reduce *Np*_ac_ and, hence, N demand for leaf construction thereby increasing *PNUE*. The increase in *k*_3_ with *PPFD* tends to compensate for the direct positive effect of *PPFD* on *Np*_ac_, thereby lowering N demand for leaf construction under high light environments. Similarly, the decrease of *k*_3_ with *T*_g_ mitigates the direct negative effect of temperature on *Np*_ac_, thereby equalizing the N demand for a range of temperature. Mostly independently from changes in *k*_3_ (since these two traits are not correlated across plant species), *J*_fac_ increases with *C*_a_, in agreement with the lower decline under elevated CO_2_ of *J*_max_ compared to 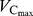 [33]. Moreover, *J*_fac_ is negatively related to *PPFD*, which is in good agreement with the higher allocation of leaf N to chlorophyll observed in low *PPFD* acclimation experiments [57]. Like the increase in *k*_3_, the decrease in *J*_fac_ with *PPFD* tends to compensate for the direct positive effect of *PPFD* on *Np*_ac_, especially for species with low *k*_3_ value. Finally, the effect of temperature on *J*_fac_ is not significant which is in agreement with previous studies that reports constant *J*_fac_ with temperature (e.g. [33]).

### Uncertainties in the Calculation of the Coordinated Leaf Photosynthetic N Content

Our model takes into account the two main biochemical processes controlling leaf photosynthesis as well as the biophysical process controlling stomatal conductance. Recently, leaf mesophyll conductance has also been identified as an important biophysical limitation of photosynthesis [58]–[60], particularly for species with low *SLA* by decreasing 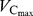 more than *J*_max_ [61], [62] and particularly during plant acclimation to water stress condition [58], [59]. Applying mesophyll conductance in our model would first require recalculating 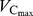 parameter from a non-rectangular hyperbola of the *A*_n_-*C*_i_ curve and with a new set of Rubisco kinetic constants, for example [58]. Moreover, it would also require the incorporation in our model of the CO_2_ diffusion mechanism between intercellular and chloroplast spaces according to a mesophyll conductance parameter [59], [60]. Furthermore, the coupling between *A*_n_ and *g*_s_ leading to the calculation of *A*_n_ would require solving a new system of equations and unknowns. Finally, this would require additional mesophyll conductance data, which were not available in our dataset. The inclusion of a variable mesophyll conductance [61], [62], as well as of other mechanisms implied in plant responses to water deficits [63], would allow testing the photosynthetic coordination hypothesis under severe abiotic stress conditions. With the coordination model reported here that does not include these processes, *N*_a_ values are lower than *N*_ac_ values under more severe abiotic stress conditions (data not shown).

The calculation of *Np*_ac_ relies on a number of plant parameter and environmental variables, leading to further uncertainties (see Text S1, Table S2 and Fig. S2–S3 for full details). Apart from *SLA*, *k*_3_ and *J*_fac_, all plant parameters were assumed to have a single set of values across the entire dataset (Table 2). Since the photosynthetic model was shown to be little sensitive to most of these parameters (Text S1, Fig. S3), using species-specific values would only marginally increase the accuracy of *N*_a_ prediction.

### Implications

Overall, our study confirms the basic assumption of the coordination hypothesis: leaves coordinate the development of 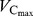 and *J*_max_ such that *W*_c_ equals *W*_j_. This opens opportunities to couple C and N at a global scale by incorporating the coordination hypothesis into dynamic global vegetation models (DGVMs). However, the applicability of this hypothesis for improved prediction of photosynthetic capacity and leaf nitrogen content depends on the accuracy at which we can determine key parameters of the combined photosynthesis - stomatal conductance – leaf N model as well as the timescale of plant regulatory photosynthesis mechanisms. The two key parameters *J*_fac_ and *k*_3_ seem to be predictable from a combination of environmental growth conditions - probably due to the strong dependence of the development of the photosynthetic machinery on environment variables – and information about plant growth form or PFT. However, the morphological trait *SLA* does not seems to be predictable with sufficient accuracy from environmental conditions which is consistent with the large functional diversity found in a given environment [64]. *SLA* needs to be defined at least by PFT and preferably by species. This study thus confirms the relevance of leaf morphology, represented by *SLA*, in photosynthesis, which has been pointed out before, (*e.g.* [56]). However, *SLA* is one of the best-studied plant traits worldwide (*e.g.* [36]) and it may be possible to determine *SLA* with sufficient accuracy for a large range of C_3_ species. Finally, although the turnover of photosynthetic enzymes like Rubisco can be seen as very constrained within the C_3_ plant kingdom, to our knowledge there is no study that investigates its variability across species. We therefore stress the need for further comparative research quantifying the variability of photosynthetic enzyme turnover across C_3_ species. Further tests of the coordination hypothesis will require, during plant growth, coupled measurements of microclimate, of leaf gas exchanges and of photosynthetic traits, including the dynamics of Rubisco, within the canopy [65].

### Conclusion

This study bridges a gap concerning the coupling of C and N fluxes in C_3_ plant species. It confirms the basic assumption of the leaf photosynthesis coordination hypothesis and demonstrates that this hypothesis can be successfully applied across species and PFTs and under a wide range of climates. Moreover, we have shown that *k*_3_ and *J*_fac_ in combination with *SLA* are major plant functional traits, which reflect plant adaptation to light, temperature and N availability during growth. Surprisingly, few studies provide both leaf photosynthetic parameters and environmental conditions during plant growth. Improved datasets combining the *k*_3_ and *J*_fac_ photosynthetic traits with the *SLA* morphological trait are needed to further increase our understanding of leaf economics (C–N stœchiometry) and plant strategies. The leaf photosynthesis coordination model reported here has been successfully used in a patch scale grassland vegetation model [66], [67]. Further applications include modeling at regional and global scales the role of plant diversity for the carbon and nitrogen cycles.

## Supporting Information

Figure S1 **Details on the leaf photosynthesis coordination hypothesis.** Variation of leaf carboxylation rates with leaf nitrogen content for three levels of radiations (A–C). According to the leaf photosynthesis coordination theory, a leaf photosynthetic N content is determined as colimiting the carboxylation/oxygenation of ribulose-1,5-bisphosphate (RuBP) by the enzyme ribulose 1·5-bisphosphate carboxylase/oxygenase (Rubisco; *W*_c_), and the regeneration of RuBP by the electron transport chain (*W*_j_). Below *N*_pac_, the photosynthesis will be limited by the Rubisco activity and therefore by the amount of leaf proteins. Beyond *N*_pac_, the marginal gain of photosynthesis per unit of leaf proteins is weak. Along the vertical canopy profile, *N*_pac_ declines with transmitted radiation when all other variables are equal.
(TIF)

Figure S2
**Mean temperature functions of the maximum rates of carboxylation (** 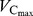 **) and electron transport (** ***J*** **_max_) and their ratio (** 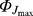 **/** 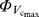 **).** Functions were calculated using the parameters related to temperature sensitivity (activation and deactivation enthalpies and entropy) as calibrated by Kattge & Knorr (2007) for many species (48 species for 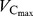, 32 for *J*_max_ and 29 for their ratio). The error bars correspond to the standard errors among species representing the inter-specific variability.(TIF)

Figure S3 **Sensitivity analysis of the photosynthesis-stomatal conductance model.** Following Félix & Xanthoulis (2005), a sensitivity analysis of the models calibrated for *Dactylis glomerata* with common one-to-one variation of parameters (±15%). Output variables are shown as lines, parameters as columns. The sensitivity index (IOS) was calculated as the maximal ratio of output variation to parameter variation during a climatic scenario (air temperature, *PPFD*, *h*_s_ and *C*_a_) recorded from an upland site in central France (Theix, 45°43′N, 03°01′E, 870 m) for years 2003–2004. Color tones indicate sensitivity index (positive, red; negative, blue).
(TIF)

Table S1 **Dataset used for the validation of leaf photosynthesis coordination.** The excel file includes the leaf photosynthetic parameters and the environmental growth conditions used to calculate *W*_c_, *W*_j_ and *N*_ac_.
(XLS)

Table S2 **Range of the observed values among literature of the parameters used in the leaf photosynthesis – stomatal conductance model.** The categories were the minimum, the maximum, the median and the percentage of variation of parameters range. The sources of observations were also reported. The sources, where the minimum and maximum values were observed, were annotated with – and +. A reference temperature of 20°C was used.
(DOC)

Table S3 **Multiple regression analyses of** ***Vc*** **_max_ and** ***J*** **_max_ from environmental growth conditions for the bootstrap analysis.** Independent variables: X_1_: air CO_2_ concentration (*C*_g_); X_2_: N level; X_3_: soil H_2_O level; X_4_: radiation (*PPFD*); X_5_: air growth temperature (*T*_g_); X_6_: air relative humidity (*h*_s_). The number of observations was 236.
(DOC)

Table S4 **Multiple regression analyses of** ***k*** **_3_ and** ***J*** **_fac_ from environmental growth conditions for a bootstrap analysis.** Independent variables were the same as Table S3. The number of observations was 236.
(DOC)

Table S5 **Prediction of** ***W*** **_c_ and** ***W*** **_j_ (µmol m^−2^ s^−1^) in using the parameters** ***Vc*** **_max_ and** ***J*** **_max_ calculated from regression analyses on the independent part of the dataset in a bootstrap analysis (Table S3).** Characteristics of the *W*_c/_ *W*_j_ relationship. The intercepts of regression for each PFT were set to zero (since there were not significantly different from zero) to estimate the slopes. RRMSE: relative root mean square error.
(DOC)

Table S6 **Prediction of** ***N*** **_ac_ in using the parameters** ***k*** **_3_ and** ***J*** **_fac_ calculated from the regression analyses on the independent part of the dataset in a bootstrap analysis (Table S4).** Characteristics of the relationship between predicted and observed leaf N content (*N*_ac_/*N*_a_, gN****m**^−^** ^2^). The intercepts of regression for each PFT were set to zero (since there were not significantly different from zero) to estimate the slopes. Abbreviation: RRMSES and RRMSEU are systematic and unsystematic relative root mean square error, respectively.
(DOC)

Table S7
**Dependence of leaf photosynthetic parameters on plant functional type (PFT).** ANOVA model and mean comparison test by LSD method of the PFT effect on leaf photosynthetic traits used in the test of coordination hypothesis (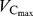, *J*_max_, *k*_3_, *J*_fac_ and *SLA*). The values of *k*_3_ and *J*_fac_ were log-transformed and all residuals followed a normal distribution. For a given variable, PFTs with the same letter belong to the same group.(DOC)

Text S1 **Sensitivity analysis of the photosynthesis – stomatal conductance model.**
(DOC)

Text S2 **Demonstration of the formalism of the coordinated leaf photosynthetic N content.**
(DOC)
