# Supplementary figures and images for: The Coordination of Leaf Photosynthesis Links C and N Fluxes in C3 Plant Species
Source: PLoS One. 2012 Jun 7;7(6):e38345. doi: 10.1371/journal.pone.0038345 (PMC3369925; doi:10.1371/journal.pone.0038345)

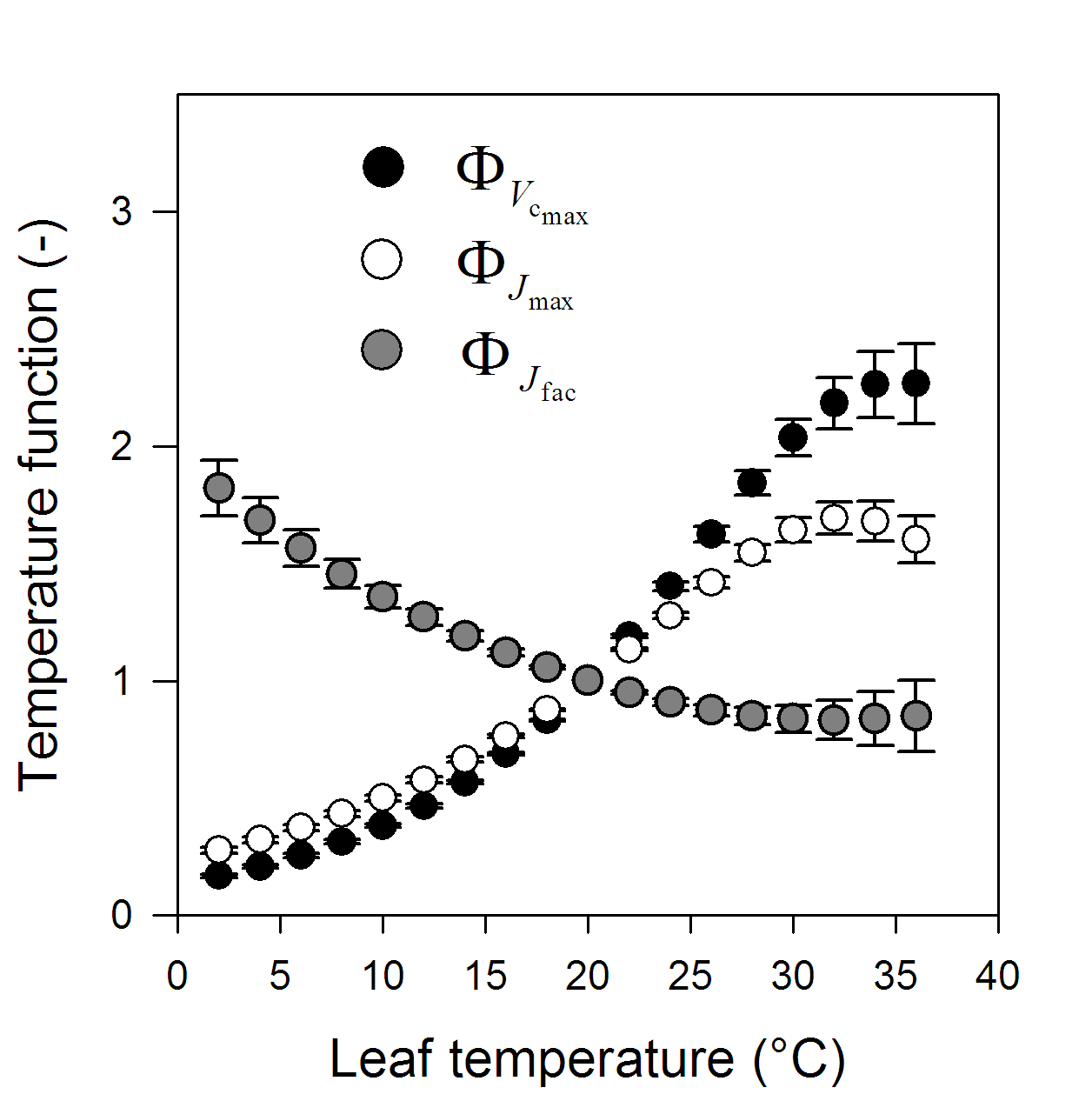

Supplement: Figure S2 — Mean temperature functions of the maximum rates of carboxylation ( ) and electron transport ( J max) and their ratio ( / ). Functions were calculated using the parameters related to temperature sensitivity (activation and deactivation enthalpies and entropy) as calibrated by Kattge & Knorr (2007) for many species (48 species for , 32 for J max and 29 for their ratio). The error bars correspond to the standard errors among species representing the inter-specific variability. (TIF) [file pone.0038345.s002.tif]

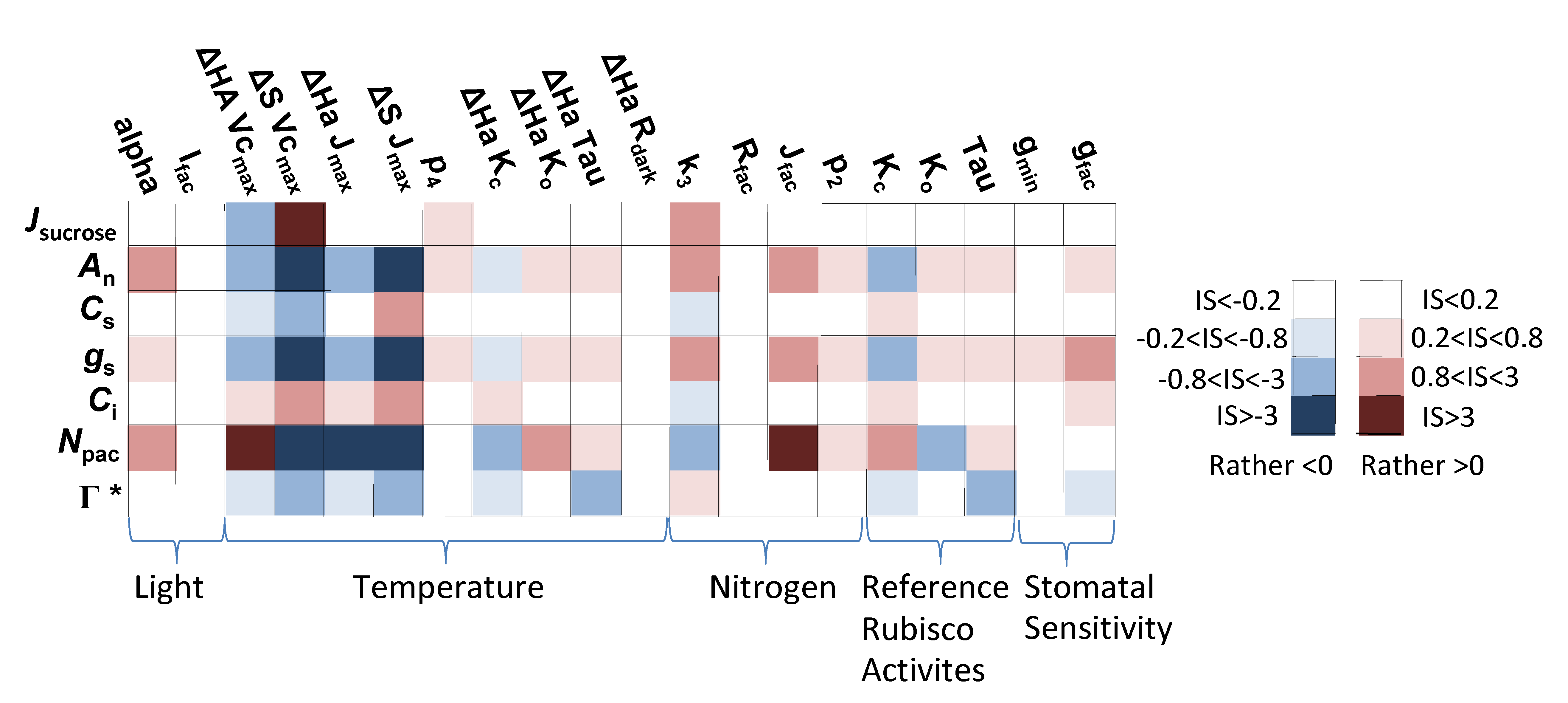

Supplement: Figure S3 — Sensitivity analysis of the photosynthesis-stomatal conductance model. Following Félix & Xanthoulis (2005), a sensitivity analysis of the models calibrated for Dactylis glomerata with common one-to-one variation of parameters (±15%). Output variables are shown as lines, parameters as columns. The sensitivity index (IOS) was calculated as the maximal ratio of output variation to parameter variation during a climatic scenario (air temperature, PPFD, h s and C a) recorded from an upland site in central France (Theix, 45°43′N, 03°01′E, 870 m) for years 2003–2004. Color tones indicate sensitivity index (positive, red; negative, blue). (TIF) [file pone.0038345.s003.tif]
