# Supplementary material for: The Coordination of Leaf Photosynthesis Links C and N Fluxes in C3 Plant Species
Source: PLoS One. 2012 Jun 7;7(6):e38345. doi: 10.1371/journal.pone.0038345 (PMC3369925; doi:10.1371/journal.pone.0038345)
Supplement: Table S2 — Range of the observed values among literature of the parameters used in the leaf photosynthesis – stomatal conductance model. The categories were the minimum, the maximum, the median and the percentage of variation of parameters range. The sources of observations were also reported. The sources, where the minimum and maximum values were observed, were annotated with – and +. A reference temperature of 20°C was used. (DOC) [file pone.0038345.s005.doc]

**Table S2**: Range of the observed values among literature of the parameters used in the leaf photosynthesis – stomatal conductance model. The categories were the minimum, the maximum, the median and the percentage of variation of parameters range. The sources of observations were also reported. The sources, where the minimum and maximum values were observed, were annotated with – and +. A reference temperature of 20°C was used.

| **Parameter** | **Minimum** | **Maximum** | **Median** | **% variation** | **Source** |
| --- | --- | --- | --- | --- | --- |
| Α | 0.015 | 0.09 | 0.05 | 71.4 | 10^-^, 16, 22, 27^+^, 38 |
| *I*_fac_ | - | - | 0.5 | - | 10 |
| ∆Ha *V*c_max_ | 45 371 | 149 061 | 66 437 | 53.3 | 4-5, 9-10, 15, 19, 24-26, 28-29, 36-37^-/+^ |
| ∆S *V*c_max_ | 606 | 667 | 648 | 4.8 | 10, 19^-^, 28, 37^+^ |
| ∆Ha *J*_max_ | 34 800 | 115 191 | 50 900 | 53.6 | 4, 9-10, 15, 19, 26, 28-29, 35-36^+^, 39 |
| ∆S *J*_max_ | 630 | 659 | 645 | 2.2 | 10, 19, 28, 37^-/+^ |
| *p*_4_ | 0.81 | 1.07 | 0.94 | 13.8 | 19^-/+^ |
| *p_2_* | 0.026 | 0.044 | 0.036 | 25.7 | 1^-/+^ |
| ∆Ha *K*_c_ | 59 536 | 80 470 | 67 900 | 15.0 | 2^-^, 5, 10, 15-16, 18, 24, 28-29^+^ |
| ∆Ha *K*_o_ | 14 510 | 36 380 | 35 974 | 43.0 | 2^-^, 5, 10, 15-16, 18, 24, 28-29^+^ |
| ∆Ha_τ_ | -28 990 | -29 000 | -28 990 | 0.01 | 10^-^, 15^+^, 16^-^, 18^+^, 29^-^ |
| ∆Ha *R*_dark_ | 9 593 | 66 405 | 38 000 | 74.8 | 4, 9-10, 15, 24^+^, 28, 36^-^ |
| *R*_fac_ | 0.0048 | 0.1010 | 0.0241 | 90.9 | 9, 20, 24, 27^-^, 28, 38^+^ |
| *K*_c_ | 15.55 | 31.83 | 20.19 | 34.4 | 5-7, 10, 14-15^+^, 18, 21-22, 24, 28-29^-^, 32 |
| *K*_o_ | 12 097 | 38 008 | 28 522 | 51.7 | 5-7, 10, 14-15^+^,18, 21-22, 24, 26, 32, |
| τ | 2 834 | 3 309 | 2 838 | 7.7 | 10, 15^-^-16, 18^+^, 28 |
| *g*_min_ | 11.7 | 193.1 | 70 | 88.6 | 3-4, 15, 20, 38^-/+^ |
| *g*_fac_ | 6.5 | 24.7 | 11.4 | 58.3 | 3-4, 10^-^, 15, 20, 38^+^ |

**References**

1. Ainsworth, E.A. & Long, S.P. (2005) What have we learned from 15 years of free-air CO_2_ enrichment (FACE)? A meta-analytic review of the responses of photosynthesis canopy. *New Phytologist*, **165**, 351-371.

2. Badger, M.R. & Collatz, G.J. (1977) Studies on the kinetic mechanism of ribulose-1,5-bisphosphate carboxylase and oxygenase reactions, with particular reference to the effect of temperature on kinetic parameters. Canergie Institution of Washington, Year Book, **76**, 355-361.

3. Baldocchi, D.D. (1994) An analytical solution for coupled leaf photosynthesis and stomatal conductance models. *Tree Physiology*, **14**: 1069-1079.

4. Baldocchi, D.D. & Harley, P.C. (1995) Scaling carbon-dioxide and water-vapour exchange from leaf to canopy in a deciduous forest .2. Model testing and application. *Plant, Cell and Environment*, **18**, 1157-1173.

5. Bernacchi, C.J., Singsaas, E.L., Pimentel, C., Portis, A.R. & Long, S.P. (2001) Improved temperature response functions for models of Rubisco-limited photosynthesis. *Plant, Cell and Environment*, **24**, 253-259.

6. Caemmerer, S.v., Evans, J.R., Hudson, G.S. &, Andrews, T.J. (1994) The kinetics of ribulose-1,5-bisphosphate carboxylase / oxygenase in vivo inferred from measurements of photosynthesis in leaves of transgenic tobacco. *Planta*, **195,** 88-97.

7. De Pury, D.G.G. & Farquhar, G.D. (1997) Simple scaling of photosynthesis from leaves to canopies without the errors of big-leaf models. *Plant, Cell and Environment*, **20**, 537-557.

8. Ehleringer, J. & Bjorkman, O. (1977) Quantum yields for CO_2_ uptake in C_3_ and C_4_ plants. Dependence on temperature, CO_2_ and O_2_ concentration. *Plant Physiology*, **59**, 86-90.

9. Dreyer, E., LeRoux, X., Montpied, P., Daudet, F.A. & Masson, F. (2001) Temperature response of leaf photosynthetic capacity in seedlings from seven temperate tree species. *Tree Physiology*, **21**, 223-232.

10. Falge, E., Graber, W., Siegwolf, R. & Tenhunen, J.D. (1996) A model of the gas exchange response of *Picea abies* to habitat conditions. *Trees-Struct Function*, **10**, 277-287.

11. Farquhar, G.D., Caemmerer, S.v. & Berry, J.A. (1980) A biochemical model of photosynthetic CO_2_ assimilation in leaves of C_3_ species. *Planta*, **149**, 78-90.

12. Félix, R. & Xanthoulis, D. (2005) Sensitivity analysis of the mathematical model "Erosion Productivity Impact Calculator" (EPIC) by approach One-factor-at-a-time (OAT). Biotech *Agronomy, Society and Environment*, **9**, 179-190.

13. Friend, A.D. (2001) Modelling canopy CO_2_ fluxes: are 'big-leaf' simplifications justified? *Global Ecol Biogeography*, **10**, 603-619.

14. Friend, A.D. (1991) Use of a model of photosynthesis and leaf microenvironment to predict optimal stomatal conductance and leaf nitrogen partitioning. *Plant, Cell and Environment*, **14**, 895-905.

15. Harley, P.C. & Baldocchi, D.D. (1995) Scaling carbon-dioxide and water-vapor exchange from leaf to canopy in a deciduous forest .1. Leaf model parametrization. *Plant, Cell and Environment*, **18**, 1146-1156.

16. Harley, P.C. & Tenhunen, J.D. (1991) Modeling the photosynthetic response of C_3_ leaves to environmental factors. *Modeling crop photosynthesis from biochemistry to canopy*. CSSA special publication 19.

17. Jones, H.G. (1992) Plants and microclimate. In: *A quantitative approach to environmental physiology*. 2^nd^ Edition. Cambridge University Press, Cambridge 426 p

18. Jordan, D.B. & Ogren, W.L. (1984) The CO_2_/O_2_ specificity of ribulose 1,5-bisphosphate carboxylase / oxygenase. Dependence on ribulosebisphosphate concentration, pH and temperature. *Planta*, **161**, 308-313.

19. Kattge, J. & Knorr, W. (2007) Temperature acclimation in a biochemical model of photosynthesis: a reanalysis of data from 36 species. *Plant, Cell and Environment*, **30**, 1176-1190.

20. Kim, S.H. & Lieth, J.H. (2003) A coupled model of photosynthesis, stomatal conductance and transpiration for a rose leaf (*Rosa hybrida L.*). *Annals of Botany*, **91**, 771-781.

21. Kirschbaum, M.U.F. & Farquhar, G.D. (1984) Temperature dependence of whole-leaf photosynthesis in *Eucalyptus pauciflora Sieb. ex Spreng*. *Australian Journal of Plant Physiology*, **11**, 519-538.

22. Kull, O. & Kruijt, B. (1998) Leaf photosynthetic light response: a mechanistic model for scaling photosynthesis to leaves and canopies. *Functional Ecology*, **12**, 767-777.

23. Leuning, R., Kelliher, F.M., Depury, D.G.G. & Schulze E.D. (1995) Leaf nitrogen, photosynthesis, conductance and transpiration - scaling from leaves to canopies. *Plant, Cell and Environment*, **18**, 1183-1200.

24. Long, S.P. (1991) Modification of the response of photosynthetic productivity to rising temperature by atmospheric CO_2_ concentrations: has its importance been underestimated? *Plant, Cell and Environment*, **14**, 729-739.

25. Long, S.P., Ainsworth, E.A., Rogers, A. & Ort, D.R. (2004) Rising atmospheric carbon dioxide: Plants face the future. *Annual Review of Plant Biology*, **55**, 591-628.

26. Medlyn, B.E., Dreyer, E., Ellsworth, D., Forstreuter, M., Harley, P.C., Kirschbaum, M.U.F., Le Roux, X., Montpied, P., Strassemeyer, J., Walcroft, A. & et al. (2002) Temperature response of parameters of a biochemically based model of photosynthesis. II. A review of experimental data. *Plant, Cell and Environment*, **25**, 1167-1179.

27. Meir, P., Kruijt, B., Broadmeadow, M., Barbosa, E., Kull, O., Carswell, F., Nobre, A. & Jarvis, P.G. (2002) Acclimation of photosynthetic capacity to irradiance in tree canopies in relation to leaf nitrogen concentration and leaf mass per unit area. *Plant, Cell and Environment*, **25**, 343-357.

28. Muller, J., Wernecke, P. & Diepenbrock, W. (2005) LEAFC_3_-N: a nitrogen-sensitive extension of the CO_2_ and H_2_O gas exchange model LEAFC_3_ parameterised and tested for winter wheat (*Triticum aestivum L.*). *Ecological Modelling*, **183**, 183-210.

29. Niinemets, U. & Tenhunen, J.D. (1997) A model separating leaf structural and physiological effects on carbon gain along light gradients for the shade-tolerant species *Acer saccharum*. *Plant, Cell and Environment,* **20**, 845-866.

30. Nikolov, N.T., Massman, W.J. & Schoettle, A.W. (1995) Coupling biochemical and biophysical processes at the leaf level - an equilibrium photosynthesis model for leaves of C_3_ plants. *Ecological Modelling*, **80**, 205-235.

31. Rolland, F., Moore, B. & Sheen, J. (2002) Sugar sensing and signaling in plants. *Plant Cell*, **14**, S185-S205.

32. Sage, R.F. & Sharkey, T.D. (1987) The effect of temperature on the occurrence of O_2_ and CO_2_ insensitive photosynthesis in field grown plants. *Plant Physiology*, **84**, 658-664.

33. Sinoquet, H., Rakocevic, M. & Varlet-Grancher, C. (2000) Comparison of models for daily light partitioning in multispecies canopies. *Agriculture, Forest and Meteorology*, **101**, 251-263.

34. Suzuki, Y., Makino, A. & Mae, T. (2001) Changes in the turnover of Rubisco and levels of mRNAs of rbcL and rbcS in rice leaves from emergence to senescence. *Plant, Cell and Environment*, **24**, 1353-1360.

35. Tcherkez, G.G.B., Farquhar, G.D. & Andrews, T.J. (2006) Despite slow catalysis and confused substrate specificity, all ribulose bisphosphate carboxylases may be nearly perfectly optimized. *Proceedings of the National Academy of Sciences of the united states of America*, **103**, 7246-7251.

36. Walcroft, A.S., Whitehead, D., Silvester, W.B. & Kelliher, F.M. (1997) The response of photosynthetic model parameters to temperature and nitrogen concentration in *Pinus radiata D.Don*. *Plant, Cell and Environment*, **20**, 1338-1348.

37. Wohlfahrt, G., Bahn, M., Haubner, E., Horak, I., Michaeler, W., Rottmar, K., Tappeiner, U. & Cernusca, A. (1999) Inter-specific variation of the biochemical limitation to photosynthesis and related leaf traits of 30 species from mountain grassland ecosystems under different land use. *Plant, Cell and Environment*, **22**, 1281-1296.

38. Wohlfahrt, G., Bahn, M., Horak, I., Tappeiner, U. & Cernusca, A. (1998) A nitrogen sensitive model of leaf carbon dioxide and water vapour gas exchange: application to 13 key species from differently managed mountain grassland ecosystems. *Ecological Modelling*, **113**, 179-199.

39. Wullschleger, S.D. (1993) Biochemical limitations to carbon assimilation in C_3_ plants - A retrospective analysis of the A/C_i_ curves from 109 species. *Journal of Experimental Botany*, **44**, 907-920.

40. Yin, X., Van Oijen, M. & Schapendonk, A. (2004) Extension of a biochemical model for the generalized stoichiometry of electron transport limited C_3_ photosynthesis. *Plant, Cell and Environment* **27**, 1211-1222.
