# Supplementary material for: The Coordination of Leaf Photosynthesis Links C and N Fluxes in C3 Plant Species
Source: PLoS One. 2012 Jun 7;7(6):e38345. doi: 10.1371/journal.pone.0038345 (PMC3369925; doi:10.1371/journal.pone.0038345)
Supplement: Table S3 — Multiple regression analyses of Vc max and J max from environmental growth conditions for the bootstrap analysis. Independent variables: X1: air CO2 concentration (C g); X2: N level; X3: soil H2O level; X4: radiation (PPFD); X5: air growth temperature (T g); X6: air relative humidity (h s). The number of observations was 236. (DOC) [file pone.0038345.s006.doc]

**Table S3 Multiple regression analyses of *Vc*_max_ and *J*_max_ from environmental growth conditions for the bootstrap analysis.** Independent variables: X_1_: air CO_2_ concentration (*C*_g_); X_2_: N level; X_3_: soil H_2_O level; X_4_: radiation (*PPFD*); X_5_: air growth temperature (*T*_g_); X_6_: air relative humidity (*h*_s_). The number of observations was 236

|  | *Vc*_max_ | | |  | *J*_max_ | | |
| --- | --- | --- | --- | --- | --- | --- | --- |
| Data | Equation | *P-value* | *r^2^* |  | Equation | *P-value* | *r^2^* |
| 1 | Y = -29.9-8.32*X_1_+3.53*X_2_-23.1*X_3_+0.25*X_4_-2.60*X_5_+236.6*X_6_-0.33*X_4_*X_6_ | < 0.001 | 0.66 |  | Y = -78.3-6.95*X_1_+9.03*X_2_-65.6*X_3_+0.66*X_4_-12.7*X_5_+763.7*X_6_-1.09*X_4_*X_6_+0.01*X_4_*X_5_ | < 0.001 | 0.63 |
| 2 | Y = 5.18-8.57*X_1_+3.55*X_2_-15.6*X_3_+0.22*X_4_-3.23*X_5_+184.0*X_6_-0.29*X_4_*X_6_ | < 0.001 | 0.62 |  | Y = 98.6- 5.99*X_1_+9.36*X_2_-34.6*X_3_+0.46*X_4_-23.0*X_5_+693.0*X_6_-1.12*X_4_*X_6_+0.02*X_4_*X_5_ | < 0.001 | 0.58 |
| 3 | Y = -13.4-6.71*X_1_+4.29*X_2_-16.5*X_3_+0.23*X_4_-2.98*X_5_+190.5*X_6_ -0.29*X_4_*X_6_ | < 0.001 | 0.61 |  | Y = 8.53-0.12*X_1_+11.42*X_2_-39.7*X_3_+0.54*X_4_-21.8*X_5_+761.1*X_6_-1.17*X_4_*X_6_+0.02*X_4_*X_5_ | < 0.001 | 0.57 |
| 4 | Y = -75.2-9.18*X_1_+3.92*X_2_-18.2*X_3_+0.33*X_4_-2.91*X_5_+283.6*X_6_-0.42*X_4_*X_6_ | < 0.001 | 65.2 |  | Y = -149.2-6.0*X_1_+10.7*X_2_-45.9*X_3_+0.80*X_4_-22.0*X_5_+1008.7*X_6_-1.52*X_4_*X_6_+0.02*X_4_*X_5_ | < 0.001 | 0.62 |
| 5 | Y = -56.5-8.01*X_1_+4.02*X_2_-18.6*X_3_+0.30*X_4_-2.90*X_5_+259.2*X_6_-0.39*X_4_*X_6_ | < 0.001 | 0.62 |  | Y = -112.5-4.62*X_1_+10.8*X_2_-47.45*X_3_+0.74*X_4_-20.2*X_5_+924.1*X_6_-1.40*X_4_*X_6_+0.02*X_4_*X_5_ | < 0.001 | 0.60 |
| 6 | Y = -49.6-7.10*X_1_+3.84*X_2_-19.4*X_3_+0.28*X_4_-2.96*X_5_+250.5*X_6_-0.35*X_4_*X_6_ | < 0.001 | 0.65 |  | Y = -36.1-2.67*X_1_+9.92*X_2_-44.43*X_3_+0.61*X_4_-22.7*X_5_+ 867.5*X_6_-1.29*X_4_*X_6_+0.02*X_4_*X_5_ | < 0.001 | 0.61 |
| 7 | Y = -28.13-7.64*X_1_+4.17*X_2_-19.9*X_3_+0.26*X_4_-2.92*X_5_+227.0*X_6_-0.34*X_4_*X_6_ | < 0.001 | 0.64 |  | Y = -25.2-3.69*X_1_+10.5*X_2_-50.4*X_3_+0.62*X_4_-18.0*X_5_+762.9*X_6_-1.18*X_4_*X_6_+0.01*X_4_*X_5_ | < 0.001 | 0.60 |
| 8 | Y = -18.9-7.47*X_1_+4.25*X_2_-21.2*X_3_+0.23*X_4_-2.81*X_5_+214.6*X_6_ -0.29*X_4_*X_6_ | < 0.001 | 0.64 |  | Y = -50.1-4.71*X_1_+11.6*X_2_-61.1*X_3_+0.60*X_4_-11.6*X_5_+666.9*X_6_-0.95*X_4_*X_6_+0.01*X_4_*X_5_ | < 0.001 | 0.60 |
| 9 | Y = -1.89-8.43*X_1_+4.48*X_2_-16.0*X_3_+0.23*X_4_-3.10*X_5_+186.7*X_6_- 0.30*X_4_*X_6_ | < 0.001 | 0.63 |  | Y = 84.1-4.6179*X_1_+11.8*X_2_- 36.5*X_3_+0.49*X_4_-2.60*X_5_+696.1*X_6_-1.15*X_4_*X_6_+0.02*X_4_*X_5_ | < 0.001 | 0.61 |
| 10 | Y = -46.2-6.15*X_1_+3.41*X_2_-21.1*X_3_+0.27*X_4_-2.82*X_5_+248.4*X_6_-0.34*X_4_*X_6_ | < 0.001 | 0.63 |  | Y = -111.8-1.94*X_1_+9.11*X_2_-59.9*X_3_+0.69*X_4_-12.5*X_5_+763.3*X_6_-1.10*X_4_*X_6_+0.01*X_4_*X_5_ | < 0.001 | 0.60 |
| 11 | Y = -27.7-8.72*X_1_+5.07*X_2_-21.4*X_3_+0.25*X_4_-2.60*X_5_+221.1*X_6_ -0.32*X_4_*X_6_ | < 0.001 | 0.63 |  | Y = 0.47-4.79*X_1_+12.7*X_2_-50.9*X_3_+0.56*X_4_-21.0*X_5_+800.2*X_6_-1.20*X_4_*X_6_ +0.02*X_4_*X_5_ | < 0.001 | 0.58 |
| 12 | Y = 27.0-9.95*X_1_+4.82*X_2_-13.9*X_3_+0.18*X_4_-3.28*X_5_+143.7*X_6_-0.24*X_4_*X_6_ | < 0.001 | 0.63 |  | Y = 119.7-8.97*X_1_+12.3*X_2_-34.8*X_3_+0.43*X_4_-20.2*X_5_+580.3*X_6_-0.97*X_4_*X_6_+0.02*X_4_*X_5_ | < 0.001 | 0.59 |
| 13 | Y = -11.1-7.46*X_1_+4.11*X_2_-14.24*X_3_+0.23*X_4_-3.61*X_5_+203.0*X_6_-0.31*X_4_*X_6_ | < 0.001 | 0.64 |  | Y = -43.3-5.42*X_1_+10.3*X_2_-41.1*X_3_+0.63*X_4_-15.6*X_5_+699.6*X_6_-1.08*X_4_*X_6_+0.01*X_4_*X_5_ | < 0.001 | 0.59 |
| 14 | Y = -0.38945-9.41*X_1_+4.39*X_2_-19.2*X_3_+0.22*X_4_-2.94*X_5_+193.6*X_6_-0.29*X_4_*X_6_ | < 0.001 | 0.64 |  | Y = -11.9-10.9*X_1_+11.4*X_2_-55.5*X_3_+0.59*X_4_-11.6*X_5_+616.7*X_6_-0.95*X_4_*X_6_+0.01*X_4_*X_5_ | < 0.001 | 0.59 |
| 15 | Y = -9.83-8.21*X_1_ 3.88*X_2_-19.8*X_3_+0.25*X_4_-2.71*X_5_+197.3*X_6_-0.31*X_4_*X_6_ | < 0.001 | 0.65 |  | Y = 3.25-5.93*X_1_+10.2*X_2_-53.3*X_3_+0.62*X_4_-17.5*X_5_+728.4*X_6_-1.18*X_4_*X_6_+0.01*X_4_*X_5_ | < 0.001 | 0.62 |
| 16 | Y = -21.4-7.64*X_1_+4.57*X_2_-19.0*X_3_+0.24*X_4_-2.88*X_5_+211.6*X_6_-0.31*X_4_*X_6_ | < 0.001 | 0.65 |  | Y = -36.9-6.40*X_1_+11.9*X_2_-53.7*X_3_+0.61*X_4_-13.9*X_5_+687.0*X_6_-1.04*X_4_*X_6_+0.01*X_4_*X_5_ | < 0.001 | 0.60 |
| 17 | Y = 13.1-8.40*X_1_+4.10*X_2_-15.5*X_3_+0.22*X_4_-3.28*X_5_+171.4*X_6_-0.28*X_4_*X6 | < 0.001 | 0.66 |  | Y = 121.9-4.12*X_1_+10.3*X_2_-32.5*X_3_+0.46*X_4_-26.4*X_5_+737.8*X_6_-1.22*X_4_*X_6_+0.02*X_4_*X_5_ | < 0.001 | 0.64 |
| 18 | Y = -24.9-7.80*X_1_+3.77*X_2_-17.3*X_3_+0.26*X_4_-2.93*X_5_+217.9*X_6_-0.34*X_4_*X_6_ | < 0.001 | 0.65 |  | Y = 15.7-3.92*X_1_+9.31*X_2_-35.3*X_3_+0.57*X_4_-26.10*X_5_+876.3*X_6_-1.38*X_4_*X_6_+0.02*X_4_*X_5_ | < 0.001 | 0.61 |
| 19 | Y = 18.6-8.85*X_1_+5.06*X_2_-20.9*X_3_+0.19*X_4_-2.63*X_5_+158.9*X_6_-0.24*X_4_*X_6_ | < 0.001 | 0.67 |  | Y = 13.9-9.90*X_1_+12.7*X_2_-65.7*X_3_+0.54*X_4_-7.18*X_5_+488.2*X_6_-0.76*X_4_*X_6_+0.01*X_4_*X_5_ | < 0.001 | 0.63 |
| 20 | Y = -15.9-11.2*X_1_+3.20*X_2_ -18.9*X_3_+0.26*X_4_-2.95*X_5_+222.5*X_6_-0.34*X_4_*X_6_ | < 0.001 | 0.67 |  | Y = 18.0- 12.8*X_1_+8.1*X_2_-48.1*X_3_+0.60*X_4_-19.9*X_5_+782.9*X_6_-1.22*X_4_*X_6_+0.02*X_4_*X_5_ | < 0.001 | 0.65 |
