# Supplementary material for: The Coordination of Leaf Photosynthesis Links C and N Fluxes in C3 Plant Species
Source: PLoS One. 2012 Jun 7;7(6):e38345. doi: 10.1371/journal.pone.0038345 (PMC3369925; doi:10.1371/journal.pone.0038345)
Supplement: Table S4 — Multiple regression analyses of k 3 and J fac from environmental growth conditions for a bootstrap analysis. Independent variables were the same as Table S3. The number of observations was 236. (DOC) [file pone.0038345.s007.doc]

**Table S4. Multiple regression analyses of *k*_3_ and *J*_fac_ from environmental growth conditions for a bootstrap analysis.** Independent variables were the same as Table S4. The number of observations was 236.

|  | *k*_3_ | | |  | *J*_fac_ | | |
| --- | --- | --- | --- | --- | --- | --- | --- |
| Data | Equation | *P-value* | *r^2^* |  | Equation | *P-value* | *r^2^* |
| 1 | log Y = 10^-2^.(311.2+9.12*X_1_-30.8*X_2_+19.0*X_3_+0.20*X_4_-7.64*X_5_+319.1*X_6_-0.29*X_4_*X_6_) | < 0.001 | 0.37 |  | log Y = 10^-2^.(-0.76+8.70*X_1_-0.63*X_2_-9.54*X_3_+0.10*X_4_-0.41*X_5_+162.2*X_6_-0.23*X_4_*X_6_+0.003*X_4_*X_5_) | < 0.001 | 0.50 |
| 2 | log Y = 10^-2^.(321.0+7.65*X_1_-30.3*X_2_+9.49*X_3_+0.20*X_4_-7.00*X_5_+334.9*X_6_-0.32*X_4_*X_6_) | < 0.001 | 0.35 |  | log Y = 10^-2^.( 19.6+7.71*X_1_-0.40*X_2_-5.06*X_3_+0.08*X_4_-1.11*X_5_+142.0*X_6_-0.22*X_4_*X_6_+0.004*X_4_*X_5_) | < 0.001 | 0.50 |
| 3 | log Y = 10^-2^.(301.7+18.3*X_1_-27.7*X_2_+12.0*X_3_+0.20*X_4_-7.37*X_5_+320.3*X_6_-0.29*X_4_*X_6_) | < 0.001 | 0.36 |  | log Y = 10^-2^.(-5.49+9.19*X_1_+0.002*X_2_-6.33*X_3_+0.10*X_4_-0.96*X_5_+171.4*X_6_-0.25*X_4_*X6+0.003*X_4_*X_5_) | < 0.001 | 0.51 |
| 4 | log Y = 10^-2^.(326.9+4.82*X_1_-27.4*X_2_+12.7*X3+0.19*X_4_-7.00*X_5_+312.3*X_6_-0.30*X_4_*X_6_) | < 0.001 | 0.36 |  | log Y = 10^-2^.( 3.59+9.35*X_1_+0.17*X_2_-7.64*X_3_+0.10*X_4_-1.55*X_5_+174.8*X_6_-0.26*X_4_*X_6_+0.004*X_4_*X_5_) | < 0.001 | 0.49 |
| 5 | log Y = 10^-2^.(351.8+3.90*X_1_-29.4*X_2_+12.1*X_3_+0.17*X_4_-  6.84*X_5_+ 275.9*X_6_-0.26*X_4_*X_6_) | < 0.001 | 0.33 |  | log Y = 10^-2^.(-8.75+8.77*X_1_-0.50*X_2_-6.53*X_3_+0.11*X_4_-1.53*X_5_+193.0*X_6_-0.28*X_4_*X_6_+0.004*X_4_*X_5_) | < 0.001 | 0.49 |
| 6 | log Y = 10^-2^.(326.0+8.24*X_1_-27.8*X_2_+9.11*X_3_+0.18*X_4_-6.46*X_5_+292.3*X_6_-0.25*X_4_*X_6_) | < 0.001 | 0.30 |  | log Y = 10^-2^.( 14.9+7.92*X_1_-0.53*X_2_-6.66*X_3_+0.08*X_4_-1.19*X_5_+151.9*X_6_-0.23*X_4_*X_6_+0.004*X_4_*X_5_) | < 0.001 | 0.50 |
| 7 | log Y = 10^-2^.(334.3+7.05*X_1_-30.1*X_2_+11.9*X_3_+0.20*X_4_-6.90*X_5_+304.8*X_6_-0.30*X_4_*X_6_) | < 0.001 | 0.35 |  | log Y = 10^-2^.( 8.50+8.81*X_1_-0.59*X_2_-7.26*X_3_+0.09*X_4_-0.74*X_5_+150.4*X_6_-0.22*X_4_*X_6_+0.003*X_4_*X_5_) | < 0.001 | 0.50 |
| 8 | log Y = 10^-2^.(283.0+3.58*X_1_-27.6*X_2_+5.70*X_3_+0.28*X_4_-7.40*X_5_+426.6*X_6_-0.46*X_4_*X_6_) | < 0.001 | 0.43 |  | log Y = 10^-2^.(-10.4+8.74*X_1_+0.04*X_2_-9.44*X_3_+0.11*X_4_+ 0.35*X_5_+155.1*X_6_-0.22*X_4_*X_6_+0.002*X_4_*X_5_) | < 0.001 | 0.50 |
| 9 | log Y = 10^-2^.(296.8+8.11*X_1_-25.7*X_2_+8.12*X_3_+0.24*X_4_-7.21*X_5_+371.1*X_6_-0.39*X_4_*X_6_) | < 0.001 | 0.38 |  | log Y = 10^-2^.( 17.1+9.47*X_1_-0.05*X_2_-6.62*X_3_+0.08*X_4_-0.90*X_5_+140.6*X_6_-0.22*X_4_*X_6_+0.003*X_4_*X_5_) | < 0.001 | 0.50 |
| 10 | log Y = 10^-2^.(331.9+10.1*X_1_-33.6*X_2_+8.75*X_3_+0.18*X_4_-6.03*X_5_+289.7*X_6_-0.25*X_4_*X_6_) | < 0.001 | 0.34 |  | log Y = 10^-2^.(-3.69+8.23*X_1_-0.40*X_2_-8.75*X_3_+0.10*X_4_+ 0.03*X_5_+155.1*X_6_-0.22*X_4_*X_6_+0.002*X_4_*X_5_) | < 0.001 | 0.49 |
| 11 | log Y = 10^-2^.(338.2+7.68*X_1_-25.8*X_2_+8.01*X_3_+0.17*X_4_-6.52*X_5_+281.9*X_6_-0.26*X_4_*X_6_) | < 0.001 | 0.34 |  | log Y = 10^-2^.(-6.53+9.48*X_1_-0.79*X_2_-7.49*X_3_+0.11*X_4_-0.87*X_5_+175.6*X_6_-0.25*X_4_*X_6_+0.003*X_4_*X_5_) | < 0.001 | 0.52 |
| 12 | log Y = 10^-2^.(273.8+12.3*X_1_-31.1*X_2_+17.6*X_3_+0.25*X_4_-8.00*X_5_+386.5*X_6_-0.37*X_4_*X_6_) | < 0.001 | 0.41 |  | log Y = 10^-2^.( 7.00+9.50*X_1_-0.50*X_2_-5.81*X_3_+0.09*X_4_-0.71*X_5_+149.6*X_6_-0.23*X_4_*X_6_+0.003*X_4_*X_5_) | < 0.001 | 0.50 |
| 13 | log Y = 10^-2^.(311.5+10.4*X_1_-31.3*X_2_+18.5*X_3_+0.22*X_4_-7.78*X_5_+336.7*X_6_-0.34*X_4_*X_6_) | < 0.001 | 0.35 |  | log Y = 10^-2^.(-18.8+7.42*X_1_-1.02*X_2_-5.54*X_3_+0.12*X_4_-0.78*X_5_+186.5*X_6_-0.27*X_4_*X_6_+0.003*X_4_*X_5_) | < 0.001 | 0.46 |
| 14 | log Y = 10^-2^.(323.9+10.1*X_1_-32.8*X_2_+17.6*X_3_+0.20*X_4_-7.58*X_5_+317.2*X_6_-0.31*X_4_*X_6_) | < 0.001 | 0.39 |  | log Y = 10^-2^.( 13.6+8.43*X_1_-0.52*X_2_-5.65*X_3_+0.08*X_4_-1.95*X_5_+170.4*X_6_-0.25*X_4_*X_6_+0.005*X_4_*X_5_) | < 0.001 | 0.48 |
| 15 | log Y = 10^-2^.(354.8+7.06*X_1_-25.5*X_2_+13.6*X_3_+ 0.15*X_4_-7.27*X_5_+265.5*X_6_-0.25*X_4_*X_6_) | < 0.001 | 0.35 |  | log Y = 10^-2^.( 7.49+7.80*X_1_-0.02*X_2_-8.35*X_3_+0.10*X_4_-0.71*X_5_+158.9*X_6_-0.24*X_4_*X_6_+0.003*X_4_*X_5_) | < 0.001 | 0.52 |
| 16 | log Y = 10^-2^.(355.7+6.44*X_1_-29.3*X_2_+10.0*X_3_+0.17*X_4_-7.02*X_5_+287.8*X_6_-0.27*X_4_*X_6_) | < 0.001 | 0.37 |  | log Y = 10^-2^.( 11.0+6.47*X_1_-0.35*X_2_-8.63*X_3_+0.09*X_4_+ 0.24*X_5_+133.6*X_6_-0.20*X_4_*X_6_+0.002*X_4_*X_5_) | < 0.001 | 0.50 |
| 17 | log Y = 10^-2^.(313.6+7.55*X_1_-31.3*X_2_+15.6*X_3_+0.22*X_4_-7.18*X_5_+331.3*X_6_-0.33*X_4_*X_6_) | < 0.001 | 0.36 |  | log Y = 10^-2^.( 11.9+9.26*X_1_-0.68*X_2_-4.95*X_3_+0.09*X_4_-1.89*X_5_+170.5*X_6_-0.26*X_4_*X_6_+0.004*X_4_*X_5_) | < 0.001 | 0.51 |
| 18 | log Y = 10^-2^.(291.8+11.3*X_1_-26.8*X_2_+9.31*X_3_+0.21*X_4_-6.79*X_5_+344.4*X_6_-0.32*X_4_*X_6_) | < 0.001 | 0.36 |  | log Y = 10^-2^.( 5.86+8.01*X_1_-0.80*X_2_-5.84*X_3_+0.09*X_4_-1.65*X_5_+174.6*X_6_-0.26*X_4_*X_6_+0.004*X_4_*X_5_) | < 0.001 | 0.50 |
| 19 | log Y = 10^-2^.(354.5+12.7*X_1_-27.1*X_2_+13.4*X_3_+0.13*X_4_–7.06*X_5_+226.9*X_6_-0.17*X_4_*X_6_) | < 0.001 | 0.36 |  | log Y = 10^-2^.( 19.1+8.60*X_1_-0.18*X_2_-7.51*X_3_+0.08*X_4_-1.64*X_5_+159.3*X_6_-0.24*X_4_*X_6_+0.004*X_4_*X_5_) | < 0.001 | 0.50 |
| 20 | log Y = 10^-2^.(328.1+8.46*X_1_-27.0*X_2_+12.4*X_3_+0.20*X_4_-7.45*X_5_+311.4*X_6_-0.31*X_4_*X_6_) | < 0.001 | 0.37 |  | log Y = 10^-2^.( 23.3+9.17*X_1_-0.79*X_2_-7.52*X_3_+0.08*X_4_-0.86*X_5_+138.0*X_6_-0.22*X_4_*X_6_+0.003*X_4_*X_5_) | < 0.001 | 0.52 |
