# Supplementary material for: The Coordination of Leaf Photosynthesis Links C and N Fluxes in C3 Plant Species
Source: PLoS One. 2012 Jun 7;7(6):e38345. doi: 10.1371/journal.pone.0038345 (PMC3369925; doi:10.1371/journal.pone.0038345)
Supplement: Table S5 — Prediction of W c and W j (µmol m−2 s−1) in using the parameters Vc max and J max calculated from regression analyses on the independent part of the dataset in a bootstrap analysis (Table S3). Characteristics of the W c/ W j relationship. The intercepts of regression for each PFT were set to zero (since there were not significantly different from zero) to estimate the slopes. RRMSE: relative root mean square error. (DOC) [file pone.0038345.s008.doc]

**Table S6. Prediction of *W*_c_ and *W*_j_ (µmol m^-2^ s^-1^) in using the parameters *Vc*_max_ and *J*_max_ calculated from regression analyses on the independent part of the dataset in a bootstrap analysis (Table S4).** Characteristics of the *W*_c_ / *W*_j_ relationship. The intercepts of regression for each PFT were set to zero (since there were not significantly different from zero) to estimate the slopes. RRMSE: relative root mean square error.

| **Dataset** | **# Data** | ***W*_c_** | ***W*_j_** | **Slope** | ***P-value*** | ***r^2^*** | **RRMSE** |
| --- | --- | --- | --- | --- | --- | --- | --- |
| 1 | 56 | 15.16 | 15.98 | 1.04 ±0.1 | < 0.001 | 0.93 | 0.088 |
| 2 | 59 | 15.83 | 16.59 | 1.04 ±0.1 | < 0.001 | 0.95 | 0.078 |
| 3 | 58 | 13.96 | 14.60 | 1.04 ±0.1 | < 0.001 | 0.92 | 0.078 |
| 4 | 58 | 15.68 | 15.99 | 1.00 ±0.2 | < 0.001 | 0.90 | 0.105 |
| 5 | 59 | 16.09 | 16.68 | 1.01 ±0.2 | < 0.001 | 0.92 | 0.094 |
| 6 | 58 | 15.18 | 16.24 | 1.06 ±0.1 | < 0.001 | 0.93 | 0.097 |
| 7 | 59 | 15.64 | 16.30 | 1.03 ±0.1 | < 0.001 | 0.94 | 0.084 |
| 8 | 58 | 14.59 | 15.48 | 1.06 ±0.1 | < 0.001 | 0.96 | 0.081 |
| 9 | 58 | 14.84 | 15.54 | 1.04 ±0.1 | < 0.001 | 0.96 | 0.072 |
| 10 | 57 | 15.92 | 16.72 | 1.04 ±0.1 | < 0.001 | 0.94 | 0.082 |
| 11 | 58 | 14.71 | 15.32 | 1.03 ±0.2 | < 0.001 | 0.92 | 0.095 |
| 12 | 57 | 14.77 | 15.80 | 1.06 ±0.1 | < 0.001 | 0.96 | 0.087 |
| 13 | 58 | 14.87 | 15.64 | 1.04 ±0.1 | < 0.001 | 0.94 | 0.078 |
| 14 | 59 | 14.30 | 15.28 | 1.06 ±0.1 | < 0.001 | 0.96 | 0.088 |
| 15 | 58 | 14.66 | 15.47 | 1.04 ±0.1 | < 0.001 | 0.94 | 0.087 |
| 16 | 59 | 13.93 | 14.66 | 1.05 ±0.2 | < 0.001 | 0.90 | 0.087 |
| 17 | 58 | 15.53 | 16.35 | 1.05 ±0.1 | < 0.001 | 0.95 | 0.077 |
| 18 | 57 | 14.52 | 15.52 | 1.06 ±0.1 | < 0.001 | 0.94 | 0.086 |
| 19 | 59 | 14.90 | 15.78 | 1.06 ±0.1 | < 0.001 | 0.94 | 0.085 |
| 20 | 59 | 15.65 | 16.39 | 1.03 ±0.1 | < 0.001 | 0.94 | 0.086 |
