# Supplementary material for: The Coordination of Leaf Photosynthesis Links C and N Fluxes in C3 Plant Species
Source: PLoS One. 2012 Jun 7;7(6):e38345. doi: 10.1371/journal.pone.0038345 (PMC3369925; doi:10.1371/journal.pone.0038345)
Supplement: Table S6 — Prediction of N ac in using the parameters k 3 and J fac calculated from the regression analyses on the independent part of the dataset in a bootstrap analysis (Table S4). Characteristics of the relationship between predicted and observed leaf N content (N ac/N a, gNm− 2). The intercepts of regression for each PFT were set to zero (since there were not significantly different from zero) to estimate the slopes. Abbreviation: RRMSES and RRMSEU are systematic and unsystematic relative root mean square error, respectively. (DOC) [file pone.0038345.s009.doc]

**Table S6:** **Prediction of *N*_ac_ in using the parameters *k*_3_ and *J*_fac_ calculated from the regression analyses on the independent part of the dataset in a bootstrap analysis (Table S5).** Characteristics of the relationship between predicted and observed leaf N content (*N*_ac_ / *N*_a_, gN m^-2^). The intercepts of regression for each PFT were set to zero (since there were not significantly different from zero) to estimate the slopes. Abbreviation: RRMSES and RRMSEU are systematic and unsystematic relative root mean square error, respectively.

| **Dataset** | **# Data** | ***N*_ac_** | **Slope** | ***P-value*** | ***r^2^*** | **RRMSE** | **RRMSES** | **RRMSEU** |
| --- | --- | --- | --- | --- | --- | --- | --- | --- |
| 1 | 56 | 2.06 | 0.98 (0.92-1.02) | < 0.001 | 0.84 | 0.214 | 0.023 | 0.213 |
| 2 | 59 | 1.99 | 0.96 (0.91-1.00) | < 0.001 | 0.83 | 0.192 | 0.044 | 0.201 |
| 3 | 58 | 1.89 | 0.98 (0.93-1.03) | < 0.001 | 0.46 | 0.209 | 0.021 | 0.208 |
| 4 | 58 | 1.95 | 0.96 (0.92-1.01) | < 0.001 | 0.89 | 0.203 | 0.047 | 0.211 |
| 5 | 59 | 1.93 | 0.96 (0.91-1.00) | < 0.001 | 0.74 | 0.192 | 0.043 | 0.200 |
| 6 | 58 | 2.12 | 0.99 (0.94-1.04) | < 0.001 | 0.84 | 0.201 | 0.011 | 0.200 |
| 7 | 59 | 2.03 | 0.98 (0.93-1.03) | < 0.001 | 0.83 | 0.203 | 0.022 | 0.202 |
| 8 | 58 | 1.89 | 1.00 (0.94-1.07) | < 0.001 | 0.48 | 0.262 | 0.000 | 0.262 |
| 9 | 58 | 1.76 | 0.92 (0.86-0.98) | < 0.001 | 0.51 | 0.261 | 0.085 | 0.283 |
| 10 | 57 | 2.21 | 1.02 (0.97-1.07) | < 0.001 | 0.80 | 0.192 | 0.022 | 0.200 |
| 11 | 58 | 1.92 | 0.95 (0.90-1.00) | < 0.001 | 0.66 | 0.228 | 0.052 | 0.235 |
| 12 | 58 | 1.84 | 0.97 (0.91-1.03) | < 0.001 | 0.54 | 0.252 | 0.032 | 0.251 |
| 13 | 58 | 1.82 | 1.03 (0.99-1.07) | < 0.001 | 0.74 | 0.169 | 0.033 | 0.183 |
| 14 | 59 | 1.98 | 1.00 (0.95-1.05) | < 0.001 | 0.51 | 0.206 | 0.002 | 0.206 |
| 15 | 58 | 1.88 | 0.91 (0.86-0.97) | < 0.001 | 0.61 | 0.242 | 0.091 | 0.276 |
| 16 | 59 | 1.92 | 0.97 (0.91-1.02) | < 0.001 | 0.70 | 0.234 | 0.033 | 0.234 |
| 17 | 58 | 1.97 | 1.01 (0.96-1.07) | < 0.001 | 0.59 | 0.206 | 0.011 | 0.209 |
| 18 | 57 | 1.95 | 0.97 (0.92-1.02) | < 0.001 | 0.62 | 0.211 | 0.032 | 0.212 |
| 19 | 59 | 1.98 | 0.95 (0.90-1.00) | < 0.001 | 0.54 | 0.210 | 0.052 | 0.220 |
| 20 | 59 | 1.90 | 0.95 (0.90-1.00) | < 0.001 | 0.62 | 0.243 | 0.054 | 0.250 |
