# Supplementary material for: The Coordination of Leaf Photosynthesis Links C and N Fluxes in C3 Plant Species
Source: PLoS One. 2012 Jun 7;7(6):e38345. doi: 10.1371/journal.pone.0038345 (PMC3369925; doi:10.1371/journal.pone.0038345)
Supplement: Table S7 — Dependence of leaf photosynthetic parameters on plant functional type (PFT). ANOVA model and mean comparison test by LSD method of the PFT effect on leaf photosynthetic traits used in the test of coordination hypothesis (, J max, k 3, J fac and SLA). The values of k 3 and J fac were log-transformed and all residuals followed a normal distribution. For a given variable, PFTs with the same letter belong to the same group. (DOC) [file pone.0038345.s010.doc]

**Table S7: Dependence of leaf photosynthetic parameters on plant functional type (PFT).** ANOVA model and mean comparison test by LSD method of the PFT effect on leaf photosynthetic traits used in the test of coordination hypothesis ($V_{\text{C}_{\text{max}}}$, *J*_max_, *k*_3_, *J*_fac_ and *SLA*). The values of *k*_3_ and *J*_fac_ were log-transformed and all residuals followed a normal distribution. For a given variable, PFTs with the same letter belong to the same group.

| **Variables** |  |  | ***J*_max_** | |  | $V_{\text{C}_{\text{max}}}$ | |  | ***J*_fac_** | |  | ***k*_3_** | |  | ***SLA*** | |
| --- | --- | --- | --- | --- | --- | --- | --- | --- | --- | --- | --- | --- | --- | --- | --- | --- |
|  |  |  | µmol m^-2^ s^-1^ | |  | µmol m^-2^ s^-1^ | |  | dimensionless | |  | µmol CO_2_ g^-1^ N s^-1^ | |  | m^2^ leaf g^-1^ DM | |
| **PFTs** | **Nb** |  | **Value** ± se | **Comp.** |  | **Value** ± se | **Comp.** |  | **Value** ± se | **Comp.** |  | **Value** ± se | **Comp.** |  | **Value** ± se | **Comp.** |
| PFT1 | 63 |  | 64.5 ± 4.90 | a |  | 27.7 ± 1.91 | a |  | 2.23 ± 0.03 | b |  | 46.0 ± 3.80 | a |  | 10.4 ± 0.5 | a |
| PFT2 | 48 |  | 67.5 ± 3.44 | a |  | 29.7 ± 1.72 | a |  | 2.28 ± 0.04 | b |  | 46.9 ± 6.33 | ab |  | 13.3 ± 0.4 | b |
| PFT3 | 41 |  | 115.2 ± 7.54 | b |  | 43.0 ± 2.56 | b |  | 2.51 ± 0.03 | c |  | 74.3 ± 7.74 | c |  | 21.4 ± 1.4 | d |
| PFT4 | 42 |  | 170.4 ± 9.35 | c |  | 74.7 ± 3.49 | c |  | 1.85 ± 0.02 | a |  | 85.6 ± 5.41 | c |  | 22.2 ± 0.7 | d |
| PFT5 | 66 |  | 208.2 ± 6.87 | d |  | 80.9 ± 2.38 | c |  | 2.36 ± 0.01 | c |  | 44.6 ± 0.5 | b |  | 18.6 ± 0.6 | c |
| PFT6 | 34 |  | 70.0 ± 3.44 | a |  | 27.6 ± 1.52 | a |  | 2.50 ± 0.05 | d |  | 36.2 ± 2.79 | a |  | 20.3 ± 0.9 | cd |
| Overall |  |  | *r*^2^ = 0.64, *P* < 0.001 | |  | *r*^2^ = 0.66, *P* < 0.001 | |  | *r*^2^ = 0.47, *P* < 0.001 | |  | *r*^2^ = 0.24, *P* < 0.001 | |  | *r*^2^ = 0.40, *P* < 0.001 | |
