# Supplementary material for: The Coordination of Leaf Photosynthesis Links C and N Fluxes in C3 Plant Species
Source: PLoS One. 2012 Jun 7;7(6):e38345. doi: 10.1371/journal.pone.0038345 (PMC3369925; doi:10.1371/journal.pone.0038345)
Supplement: Text S1 — Sensitivity analysis of the photosynthesis – stomatal conductance model. (DOC) [file pone.0038345.s011.doc]

**Text S1: Sensitivity analysis of the photosynthesis – stomatal conductance model**

Here a sensitivity analysis of the photosynthesis – stomatal conductance model is presented. Both range of the observed values among literature (Table S1; Fig. S2) and the systematic role of each parameter on the output variables of the model are analyzed (Fig. S3). Finally, this analysis was used to discuss on the model uncertainties linked to model calibration.

Photosynthetic variables displayed high sensitivity to parameters linked to temperature dependency of $V_{\text{C}_{\text{max}}}$ and *J*_max_ (Fig. S3), especially to their entropy (|IOS| > 3 for *A*_n_, *g*_s_, and *N*_pac_). Sensitivity to activation enthalpies was smaller but, given large variations in enthalpies across species (± 50% variation according to literature survey), this parameter was a major determinant of temperature response functions (Table S1). Activation enthalpies and entropy are, however, interdependent (Kattge & Knorr, 2007), which reduces their effects on temperature responses for air temperature lower than 30°C (Fig. S2).

The parameters *k*_3_ and *J*_fac_ (|IOS| > 0.8 for *N*_pac_, *A*_n_*,* and *g*_s_) had large effects on *A*_n_ and *N*_pac_ (Fig. S3). The parameter *R*_fac_ had no effect (Table S1), although values reported in the literature vary by ± 91% (Fig. S3). The sensitivity of the model to light was high through α (0.8 < |IOS| < 3 for *N*_pac_ and *A*_n_, and ± 71%), although *I*_fac_ had no effect. Sensitivity to the Rubisco activities was high for *K*_c_ (0.8 < |IOS| < 3 and ± 34%), intermediate for *K*_o_ (0.2 < |IOS| < 3 and ± 52%) and very low for τ. Sensitivity to stomatal aperture was low both for *g*_min_ and *g*_fac_ (0.2 < |IOS| < 0.8), but variations reported in the literature were high. Other parameters linked to temperature dependency of *K*_c_, *K*_o_, and *R*_dark_ (activation enthalpies) had more modest effects on photosynthetic variables, but the range of literature values for *K*_o_ and *R*_dark_ was high (± 43 and ± 75%).

In our study, we are mainly interested in explaining the variation of the leaf photosynthetic N content (*N*_pac_). Apart from the parameters calibrated by observation (*k*_3_, *J*_fac_ and *SLA*), the calculation of *N*_pac_ relies on a number of other plant parameters and environmental variables, leading to some uncertainties. For instance, temperature response parameters (enthalpies and entropies) were fixed at the same value for all species. Since *N*_pac_ is sensitive to these parameters (Fig. S3), species-specific calibration of temperature responses would increase prediction accuracy. However, Figure S2 shows that species-specific calibration should really increase prediction accuracy for temperature above 30°C. In the same way, moisture response parameters (*g*_fac_ and *g*_min_) are known to strongly vary across plant species (Fig. S3) and a specific calibration would also be required to improve accuracy. The small but significant effects of *T*_g_ and *h*_s_ on *N*_a_ residuals are therefore likely to be caused by variations across species in these photosynthetic parameters. Moreover, the model is sensitive to apparent quantum yield (calculated on an incident radiation basis), which has been shown to be mostly constant among C_3_ species (*ca.* 0.073 molCO_2_ mol^-1^photon, Ehleringer & Bjorkman, 1977) when determined under CO_2_ saturated conditions, although the full range of values reported in the literature is higher (Table S1). Furthermore, the model is also sensitive to the Michaelis-Menten constants for carboxylase / oxygenase activities of Rubisco, *K*_c_ and *K*_o_ respectively (Fig. S3), and to their temperature responses. However, the *K*_c_-to-*K*_o_ ratio (*i.e.* the Rubisco specificity factor) is constrained by plant evolution and is therefore conserved across C_3_ species (Suzuki *et al.*, 2001).

Uncertainties also extend to environmental conditions experienced by leaves. Even at the top of the canopy, for a given transmitted *PPFD*, absorbed *PPFD* is affected by leaf angle. Nevertheless, as shown by detailed radiative balance models (Sinoquet *et al.*, 2000), the daily amount of absorbed *PPFD* is only moderately affected by leaf angle. Moreover, leaf angles are variable in plant canopies, which tend to minimize their effect at the canopy scale (Jones, 1992). Instantaneous leaf temperature may differ by several degrees from *T*_K_, however, when integrated over several days these differences tend to vanish (Jones, 1992). Since the half-life time of Rubisco is close to one month (Suzuki *et al.*, 2001), mean *PPFD* over the month preceding the measurements was used. The time span characterizing the turnover of other photosynthetic proteins could vary according to genetic and environmental factors, but is likely to match that of Rubisco as this is a prerequisite for photosynthetic coordination, which is consistent with the Rubisco regulation involving a number of mechanisms modulated by environment that act on transcriptional, post-transcriptional, translational and post-translational events (Rolland *et al.*, 2002; Long *et al.*, 2004). Further experiments could be envisaged to determine more precisely the chronology of these regulatory events and whether there is a species dependency in relation to their phenology.

**References**

Ehleringer, J. & Bjorkman, O. (1977) Quantum yields for CO_2_ uptake in C_3_ and C_4_ plants. Dependence on temperature, CO_2_ and O_2_ concentration. *Plant Physiology*, **59**, 86-90.

Jones, H.G. (1992) Plants and microclimate. In: *A quantitative approach to environmental physiology*. 2^nd^ Edition. Cambridge University Press, Cambridge 426 p

Kattge, J. & Knorr, W. (2007) Temperature acclimation in a biochemical model of photosynthesis: a reanalysis of data from 36 species. *Plant, Cell and Environment*, **30**, 1176-1190.

Long, S.P., Ainsworth, E.A., Rogers, A. & Ort, D.R. (2004) Rising atmospheric carbon dioxide: Plants face the future. *Annual Review of Plant Biology*, **55**, 591-628.

Rolland, F., Moore, B. & Sheen, J. (2002) Sugar sensing and signaling in plants. *Plant Cell*, **14**, S185-S205.

Sinoquet, H., Rakocevic, M. & Varlet-Grancher, C. (2000) Comparison of models for daily light partitioning in multispecies canopies. *Agriculture, Forest and Meteorology*, **101**, 251-263.

Suzuki, Y., Makino, A. & Mae, T. (2001) Changes in the turnover of Rubisco and levels of mRNAs of rbcL and rbcS in rice leaves from emergence to senescence. *Plant, Cell and Environment*, **24**, 1353-1360.
