# Supplementary material for: The Coordination of Leaf Photosynthesis Links C and N Fluxes in C3 Plant Species
Source: PLoS One. 2012 Jun 7;7(6):e38345. doi: 10.1371/journal.pone.0038345 (PMC3369925; doi:10.1371/journal.pone.0038345)
Supplement: Text S2 — Demonstration of the formalism of the coordinated leaf photosynthetic N content. (DOC) [file pone.0038345.s012.doc]

**Text S2: Demonstration of the formalism of the coordinated leaf photosynthetic N content**

*Demonstration of N_pac_ formalism*

When *A*_n_ is co-limited by the RuBP carboxylation / oxygenation and RuBP regeneration, the Eqn 3 can be written as:

$\left(1-\Gamma*/C_{\text{i}}\right)\cdot W_{\text{c}}-R_{\text{day}}=\left(1-\Gamma*/C_{\text{i}}\right)\cdot W_{\text{j}}-R_{\text{day}}$ (2a)

By replacing *W*_c_ and *W*_j_ by Eqn 4 and Eqn 7, respectively, and formulating $V_{\text{C}_{\text{max}}}^{\text{r}}$ and $J_{\max}^{\text{r}}$as a function of *N*_pac_ (Eqn 6 and Eqn 9, respectively), we obtained:

$\Leftrightarrow\frac{k_{3}^{\text{ac}}\cdot N_{pac}\cdot\Phi_{V_{\mathrm{c}_{\max}}}\cdot\left(C_{\mathrm{i}}-\Gamma^{*}\right)}{C_{\mathrm{i}}+k_{2}}=\frac{4\cdot\alpha\cdot\mathrm{PPFD}}{\left(1+\frac{4^{2}\cdot\alpha^{2}\cdot\mathrm{PPFD}^{2}}{\left(J_{\mathrm{fac}}\cdot k_{3}^{\text{ac}}\cdot N_{pac}\cdot\Phi_{J_{\max}}\right)^{2}}\right)^{1/2}}\cdot\frac{\left(C_{\mathrm{i}}-\Gamma^{*}\right)}{4\cdot C_{\mathrm{i}}+8\cdot\Gamma^{*}}$ (2b)

The terms including *N*_pac_ were ascribed to one side of the equation. Then, equation was elevated to square in order to break the square root.

$\Leftrightarrow N_{pac}^{2}\cdot\left(1+\frac{4^{2}\cdot\alpha^{2}\cdot\mathrm{PPFD}^{2}}{\left(J_{\mathrm{fac}}\cdot k_{3}^{\text{ac}}\cdot N_{pac}\cdot\Phi_{J_{\max}}\right)^{2}}\right)=\left(\frac{4\cdot\alpha\cdot\mathrm{PPFD}}{k_{3}^{\text{ac}}\cdot\Phi_{V_{\mathrm{c}_{\max}}}}\cdot\frac{\left(C_{\mathrm{i}}+k_{2}\right)}{\left(4\cdot C_{\mathrm{i}}+8\cdot\Gamma^{*}\right)}\right)^{2}$ (2c)

Finally, right side of equation was simplified so that there was one solution for *N*_pac_ value:

$\Rightarrow N_{\mathrm{pac}}=\frac{4\cdot\alpha\cdot\mathrm{PPFD}}{k_{3}^{\text{ac}}}\cdot\left(\left(\frac{C_{\mathrm{i}}+k_{2}}{\left(4\cdot C_{\mathrm{i}}+8\cdot\Gamma^{*}\right)\cdot\Phi_{V_{\mathrm{c}_{\max}}}}\right)^{2}-\left(\frac{1}{J_{\mathrm{fac}}\cdot\Phi_{J_{\max}}}\right)^{2}\right)^{1/2}$ (2d)
